# Targeting the MCP‐GPX4/HMGB1 Axis for Effectively Triggering Immunogenic Ferroptosis in Pancreatic Ductal Adenocarcinoma

**DOI:** 10.1002/advs.202308208

**Published:** 2024-04-09

**Authors:** Ge Li, Chengyu Liao, Jiangzhi Chen, Zuwei Wang, Shuncang Zhu, Jianlin Lai, Qiaowei Li, Yinhao Chen, Dihan Wu, Jianbo Li, Yi Huang, Yifeng Tian, Yanling Chen, Shi Chen

**Affiliations:** ^1^ Department of Hepatobiliary Surgery and Fujian Institute of Hepatobiliary Surgery Fujian Medical University Union Hospital Fuzhou 350001 China; ^2^ Shengli Clinical Medical College of Fujian Medical University Fujian Medical University Fuzhou 350001 China; ^3^ Department of Hepatopancreatobiliary Surgery Fujian Provincial Hospital Fuzhou 350001 China; ^4^ Fujian Provincial Center for Geriatrics Fuzhou 350001 China; ^5^ Fujian Key Laboratory of Geriatrics Fuzhou 350001 China; ^6^ Center for Experimental Research in Clinical Medicine Fujian Provincial Hospital Fuzhou 350001 China

## Abstract

Induction of ferroptosis can inhibit cancer cells in vitro, however, the role of ferroptosis in treatment in vivo is controversial. The immunosuppressive cells activated by the ferroptotic tumor cells can promote the growth of residual tumor cells, hindering the application of ferroptosis stimulation in tumor treatment. In this study, a new strategy is aimed to be identified for effectively triggering immunogenic ferroptosis in pancreatic ductal adenocarcinoma (PDAC) and simultaneously stimulating antitumor immune responses. Toward this, several molecular and biochemical experiments are performed using patient‐derived organoid models and a KPC mouse model (*LSL*‐*Kras^G12D^* ^/+^, *LSL‐Trp53^R172H/+^*,* Pdx‐1‐Cre*). It is observed that the inhibition of macrophage‐capping protein (MCP) suppressed the ubiquitin fold modifier (UFM)ylation of pirin (PIR), a newly identified substrate of UFM1, thereby decreasing the transcription of *GPX4*, a marker of ferroptosis, and promoting the cytoplasmic transportation of HMGB1, a damage‐associated molecular pattern. *GPX4* deficiency triggered ferroptosis, and the pre‐accumulated cytosolic HMGB1 is released rapidly. This altered release pattern of HMGB1 facilitated the pro‐inflammatory M1‐like polarization of macrophages. Thus, therapeutic inhibition of *MCP* yielded dual antitumor effects by stimulating ferroptosis and activating antitumor pro‐inflammatory M1‐like macrophages. The nanosystem developed for specifically silencing *MCP* is a promising tool for treating PDAC.

## Graphical abstract

This study developed a nanotherapy strategy targeting macrophage‐capping protein (MCP) for effectively suppressing pancreatic ductal adenocarcinoma via dual effects: inhibition of MCP reduces the UFMylation of PIR, thereby decreasing the transcription of GPX4, and promoting the cytoplasmic transportation of HMGB1. GPX4 deficiency triggers ferroptosis, then the rapid and intense release of pre‐accumulated cytosolic HMGB1 facilitates the pro‐inflammatory M1‐like polarization of macrophages.

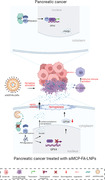

## 1 Introduction

Pancreatic ductal adenocarcinoma (PDAC) is highly malignant, with a five‐year survival rate of less than 5%.^[1]^ Systemic therapy is necessary because of limited surgical opportunities and the high probability of postoperative recurrence.^[2]^ Nevertheless, currently available systemic therapeutic regimens, including chemotherapy, radiation therapy, and immunotherapy, provide minimal satisfactory outcomes in these patients.^[^ ^3^, ^4^ ^]^

Ferroptosis is a type of programmed cell death characterized by altered oxidative homeostasis and excessive lipid peroxidation.^[5]^ Emerging evidence indicates that stimulation of ferroptosis may be an effective approach for treating cancer, as certain cancer cells are not sensitive to traditional cancer therapy due to resistance to ferroptosis but are vulnerable once ferroptosis is triggered.^[^ ^6^, ^7^, ^8^ ^]^ However, some in vivo studies have reported that stimulation of ferroptosis can promote certain types of cancers.^[^ ^9^, ^10^, ^11^ ^]^ The variable characteristics of the tumor immune microenvironment stimulated by ferroptotic cancer cells may account for the inconsistent outcomes when using ferroptosis as a target for cancer therapy.^[9]^ For example, when *GPX4* is suppressed in liver cancer, ferroptotic cancer cells activate the myeloid‐derived suppressor cells to boost tumor growth,^[10]^ and induction of ferroptosis by H_2_O_2_ in pancreatic cancer cells facilitates the polarization of tumor‐promoting pro‐resolution M2‐like macrophages.^[12]^ In contrast, ferroptosis enhances the antitumor immune reaction due to inhibition of *GPX4* in triple‐negative breast cancer,^[13]^ and cancer cells undergoing ferroptosis lead to efficient antitumor immunity when vaccinated into tumor‐bearing mice.^[14]^ As ferroptosis exerts diverse effects on tumor immunity, a strategy that can specifically induce ferroptosis with antitumor immune responses in PDAC is urgently required.

Cells release endogenous adjuvants, such as damage‐associated molecular patterns (DAMPs), during immunogenic cell death (ICD), which can elicit immune responses.^[^ ^15^, ^16^ ^]^ DAMPs, such as ATP, calreticulin (CRT), and HMGB1, promote the immunoreactivity of antigen‐presenting cells (APCs).^[^ ^17^, ^18^, ^19^, ^20^, ^21^ ^]^ Moreover, the release of HMGB1 is necessary for apoptosis‐induced immunogenicity.^[22]^ Although the majority of cancer cells harbor adequate levels of DAMPs for the activation of immune responses, a small proportion of cancer treatments can successfully induce ICD.^[^ ^23^, ^24^ ^]^ The high plasticity of DAMP release, which is inducer‐dependent, may be responsible for the uncertainty of ICD induction.^[^ ^25^, ^26^, ^27^ ^]^ Therefore, the identification of a specific target that induces ferroptosis and activates ICD in cancer cells may lead to the development of novel anti‐cancer treatments.

In this study, we demonstrated for the first time that the triggering of immunogenic ferroptosis by inhibiting macrophage‐capping protein (*MCP*) results in significant therapeutic efficacy in a PDAC patient‐derived organoid (PDO) model, KPC model, and PDAC cell lines. Using single‐cell analysis, we also found that ferroptosis stimulated by *MCP* inhibition can facilitate the polarization of tumoricidal pro‐inflammatory M1‐like macrophages. We identified pirin (PIR) to be a new substrate of UFM1 and found that the inhibition of MCP reduces UFMylation and accelerates the degradation of PIR. Suppression of the MCP‐PIR axis promoted the cytoplasmic transportation of HMGB1 and reduced *GPX4* transcription, ultimately leading to the early release of HMGB1 from *GPX4*‐deficient ferroptotic PDAC cells to activate the pro‐inflammatory M1‐like macrophages. Our approach can be used to develop a novel treatment regimen for patients with PDAC, which might improve the overall survival of these patients.

## 2 Results

### 2.1 Inhomogeneity of the Immune Microenvironment in Ferroptosis

**Figure 1:**
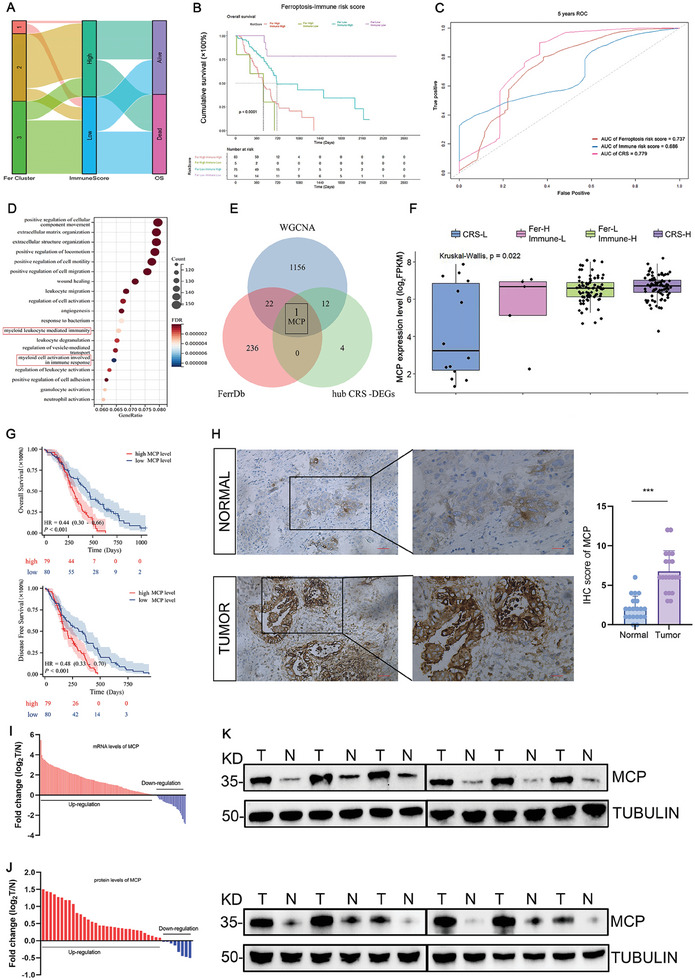
Inhomogeneity of the immune microenvironment in ferroptosis. A) Alluvial diagram of immune risk subtypes, ferroptosis clusters, and survival status. B) Kaplan−Meier analysis of overall survival based on a combination of immune and ferroptosis risk subtypes. C) The 5‐year survival rate of receiver operating characteristic (ROC) curves of different models. D) Gene Ontology analyses of the DEGs between the combined risk score‐high (CRS‐H) group and the combined risk score‐low (CRS‐L) group. E) Venn diagram of genes associated with ferroptosis and macrophages. F) MCP mRNA levels in CRS groups. G) Overall survival and disease‐free survival of patients with PDAC from Fujian Provincial Hospital with high or low levels of MCP (*n* = 159). H) Representative images and statistical analysis of MCP immunohistochemistry in pancreatic cancer tissues (*n* = 20) and adjacent normal pancreatic tissues (*n* = 20) from representative patients with PDAC (scale bar: 100 µm [left] and 50 µm [right]). I) MCP mRNA levels in pancreatic cancer tissue samples (*n* = 159) and adjacent normal pancreatic tissue samples (*n* = 159) (RT‐qPCR). J) MCP protein level in pancreatic cancer (*n* = 40) and adjacent normal pancreatic tissues (*n* = 40). K) Representative images of MCP protein levels in tumor tissues (T) and adjacent normal tissues (N) from representative patients with PDAC. n indicates individual patients. Data are shown as the mean ± SEM. P values are presented as ns *P* > 0.05, ** *P* < 0.01, ***P < 0.001. One‐way ANOVA (F,H), and log‐rank tests (B) were used.

We first collected data on patients with PDAC from The Cancer Genome Atlas (TCGA) and then constructed three ferroptosis clusters based on ferroptosis‐associated genes (FerrDb: http://www.zhounan.org/ferrdb) using unsupervised clustering analysis (Figure S1A, Supporting Information). Patients in the same ferroptosis cluster could be classified based on different immune risk scores, as determined using ESTIMATE (**Figure** **1A**), demonstrating the heterogeneity of the immune microenvironment in ferroptosis. To evaluate the impact of the immune microenvironment or ferroptosis on PDAC, we constructed ferroptosis and immune risk score models to assess the prognosis of patients with PDAC. Although each risk score alone can be used to determine patient prognosis (Figure S1B, Supporting Information), a combination of ferroptosis and immune risk scores may further stratify the prognosis based on either the ferroptosis risk score model (ferroptosis low‐immune low or ferroptosis low‐immune high) or the immune risk score model (immune high‐ferroptosis low or immune high‐ferroptosis high) (Figure 1B; Figure S1C, Supporting Information). The area under the curve (AUC) also confirmed the precision of the combined ferroptosis‐immune risk score (Figure 1C). The above results indicated that both ferroptosis and the immune microenvironment were important for patients with PDAC and that they can affect each other, thus, the combination of ferroptosis and the immune microenvironment is required for better assessment of PDAC.

Patients with the worst outcomes were classified based on the combined risk model (Figure 1B) in the “combined risk score‐high” (CRS‐H) group, while patients with the best outcomes (Figure 1B) were placed in the “combined risk score low” (CRS‐L) group. Next, we identified the differentially expressed genes (CRS‐DEGs) between these two groups (Figure S1D, Supporting Information). Gene Ontology analyses showed significant differences in immune‐related pathways, especially in pathways associated with myeloid cell activation (Figure 1D). Compared to the CRS‐L group, the CRS‐H group showed enrichment of immune checkpoint markers and immunosuppressive cells (Figure S1E,F, Supporting Information), with high infiltration and abundance of macrophages (Figure S1G, Supporting Information). These results suggested that myeloid cells, particularly macrophages, are critical for ferroptosis‐related prognosis.

To further investigate the mechanism underlying the crosstalk between the macrophages and ferroptosis in pancreatic cancer, we analyzed three gene sets and identified the most relevant genes. We first performed a weighted gene co‐expression network (WGCNA) analysis to confirm that genes from the turquoise module correlated closely with the macrophages (Figure S1H, Supporting Information). We used ferroptosis‐related genes from FerrDb as the second gene set and hub genes from the CRS‐DEGs identified using least absolute shrinkage and selection operator (LASSO) analysis (Figure S1I–K, Supporting Information) as the third gene set. Analysis of these three gene sets using a Venn diagram demonstrated that MCP was the only gene present in all three gene sets (Figure 1E). Moreover, we found that MCP was highly expressed in the CRS‐H group and showed relatively lower expression in the CRS‐L group (Figure 1F), indicating that MCP could be a hub gene that plays a crucial role in ferroptosis‐immune‐related prognosis. We found that *MCP* was expressed highly in PDAC and was associated with poor outcomes according to TCGA (Figure S1L,M, Supporting Information). Our clinical data further confirmed that patients with PDAC and high *MCP* levels had poor overall survival and disease‐free survival (Figure 1G). Furthermore, the results of immunohistochemistry (IHC) (Figure 1H), reverse transcription‐quantitative polymerase chain reaction (RT‐qPCR) (Figure 1I), and western blotting (Figure 1J,K) demonstrated that MCP expression was significantly higher in PDAC samples than in normal samples from the tumor margin tissues of the same patients.

### 2.2 MCP Prevented Ferroptosis in PDAC

**Figure 2:**
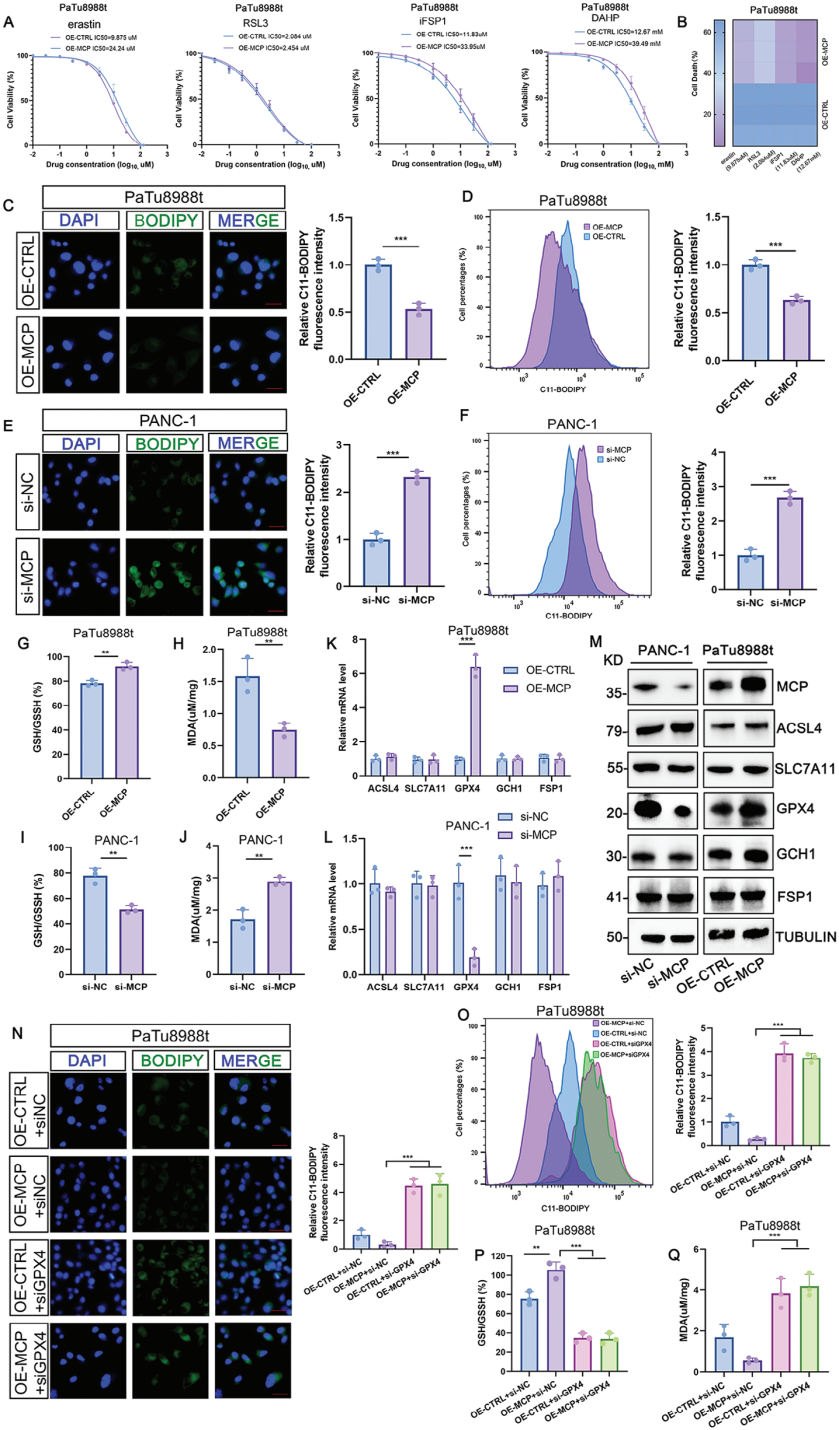
MCP prevented ferroptosis in PDAC. A) IC_50_ of the ferroptosis inducers in OE‐*CTRL* and OE‐*MCP* PaTu8988t cell lines (*n* = 3) after 48 h of treatment. B) Death of OE‐*CTRL* and OE‐*MCP* PaTu8988t cell lines treated with different ferroptosis inducers at IC_50_ identified in Figure 2A (*n* = 3). C–F) Levels of lipid peroxidation measured by fluorescence microscopy (C, E, scale bar: 30 µm) and flow cytometry (D, F), fluorescence intensity was normalized to the mean value of three replicates of the OE‐*CTRL* (C, D) and si‐*NC* (E, F) groups. G–J) Levels of GSH/GSSG ratio (G, I) and MDA (H, J) in OE‐*CTRL* and OE‐*MCP* PaTu8988t cell lines and in si‐*NC* and si‐*MCP* PANC‐1 cell lines (*n* = 3). K–M) Expression of ferroptosis‐related genes in OE‐*CTRL* and OE‐*MCP* PaTu8988t cell lines and in si‐*NC* and si‐*MCP* PANC‐1 cell lines at the mRNA (K, L) and protein (M) levels (*n* = 3). N,O, Levels of lipid peroxidation measured by fluorescence microscopy (N, scale bar: 30 µm) and flow cytometry (O), fluorescence intensity was normalized to the mean value of three replicates of the OE‐*CTRL +* si‐*NC* groups (*n* = 3). P,Q) Levels of GSH/GSSG ratio (P) and MDA (Q) in OE‐*MCP‐*PaTu8988t cells transfected with si‐*GPX4* (*n* = 3). n indicates the number of biological replicates. Data are represented as the mean ± SEM. P values are presented as ns *P* > 0.05, **P < 0.01, and ***P < 0.001. Unpaired two‐tailed Student's *t*‐test (C–L) and one‐way ANOVA (N–Q) were used.

To investigate the role of MCP in ferroptosis, we generated overexpressing (OE)‐*MCP* PaTu8988t cell lines and si‐*MCP* PANC‐1 cell lines (Figure S2A,B, Supporting Information). *MCP* upregulation accelerated cell growth, whereas *MCP* knockdown suppressed it significantly (Figure S2C, Supporting Information). Next, we detected the killing ability of several ferroptosis inducers, including erastin, RSL3, iFSP1, and DAHP, in the OE‐*CTRL* and OE‐*MCP* PaTu8988t cell lines. The half maximal inhibitory concentration (IC_50_) values of the OE‐*MCP* group were higher (**Figure** **2A**) and cell death was lower (Figure 2B) than those in the OE‐*CTRL* group. Among the ferroptosis inducers, RSL3 induced the death of the OE‐*MCP* cell line most strongly (Figure 2B). In addition, lipid peroxidation, malondialdehyde (MDA) expression, and the reduced/oxidized glutathione (GSH/GSSG) ratio were analyzed to measure ferroptosis levels. The results showed that lipid peroxidation and MDA expression decreased, whereas the GSH/GSSG ratio increased in the OE‐*MCP* group, indicating a reduction in ferroptosis level (Figure 2C,D,G,H). Conversely, increased ferroptosis was observed in the si‐*MCP* group (Figure 2E,F,I,J). In addition to the PANC‐1 and PaTu8988t cell lines, we introduced OE‐*MCP* into MIA PaCa‐2 and BxPC‐3 cell lines (the intrinsic MCP level of which was relatively low), and si‐*MCP* into Capan‐2 cell lines (the intrinsic MCP level of which was relatively high) (Figure S2A,D, Supporting Information). Consistent with the results obtained from the PANC‐1 and PaTu8988t cell lines, the cell growth was accelerated in the OE‐*MCP* group but suppressed in the the si‐*MCP* group (Figure S2E, Supporting Information). The level of ferroptosis was significantly reduced in the OE‐*MCP* group (Figure S2F,G, I,J,L,M,O,P, Supporting Information) but elevated in the si‐*MCP* group (Figure S2H,K,N,Q, Supporting Information). These results demonstrated that MCP prevented ferroptosis, whereas its downregulation triggered ferroptosis.

We measured the expression levels of several ferroptosis‐associated genes (*ACSL4*,^[28]^ *SLC7A11*,^[5]^ *GPX4*,^[29]^ *GCH1* ^[30]^, and *FSP1* ^[31]^) in OE‐*MCP* and si‐*MCP* cells to understand the mechanisms underlying MCP‐regulated ferroptosis. The results showed that both the mRNA and protein levels of GPX4 were downregulated in si‐*MCP* cells but upregulated in OE‐*MCP* cells (Figure 2K–M; Figure S2R–U, Supporting Information). To investigate the role of GPX4 in MCP‐regulated ferroptosis, we constructed si‐*GPX4* in OE‐*MCP*‐PaTu8988t cells (Figure S2V–X, Supporting Information). The results demonstrated that *GPX4* deficiency blocked the alterations in the levels of lipid peroxidation and MDA and the GSH/GSSG ratio in OE‐*MCP* cells (Figure 2N–Q). These results suggested that MCP prevented ferroptosis by upregulating GPX4.

### 2.3 Inhibition of MCP Triggered Immunogenic Ferroptosis to Suppress Pancreatic Cancer by Stimulating the Polarization of Pro‐Inflammatory M1‐Like Macrophages

**Figure 3:**
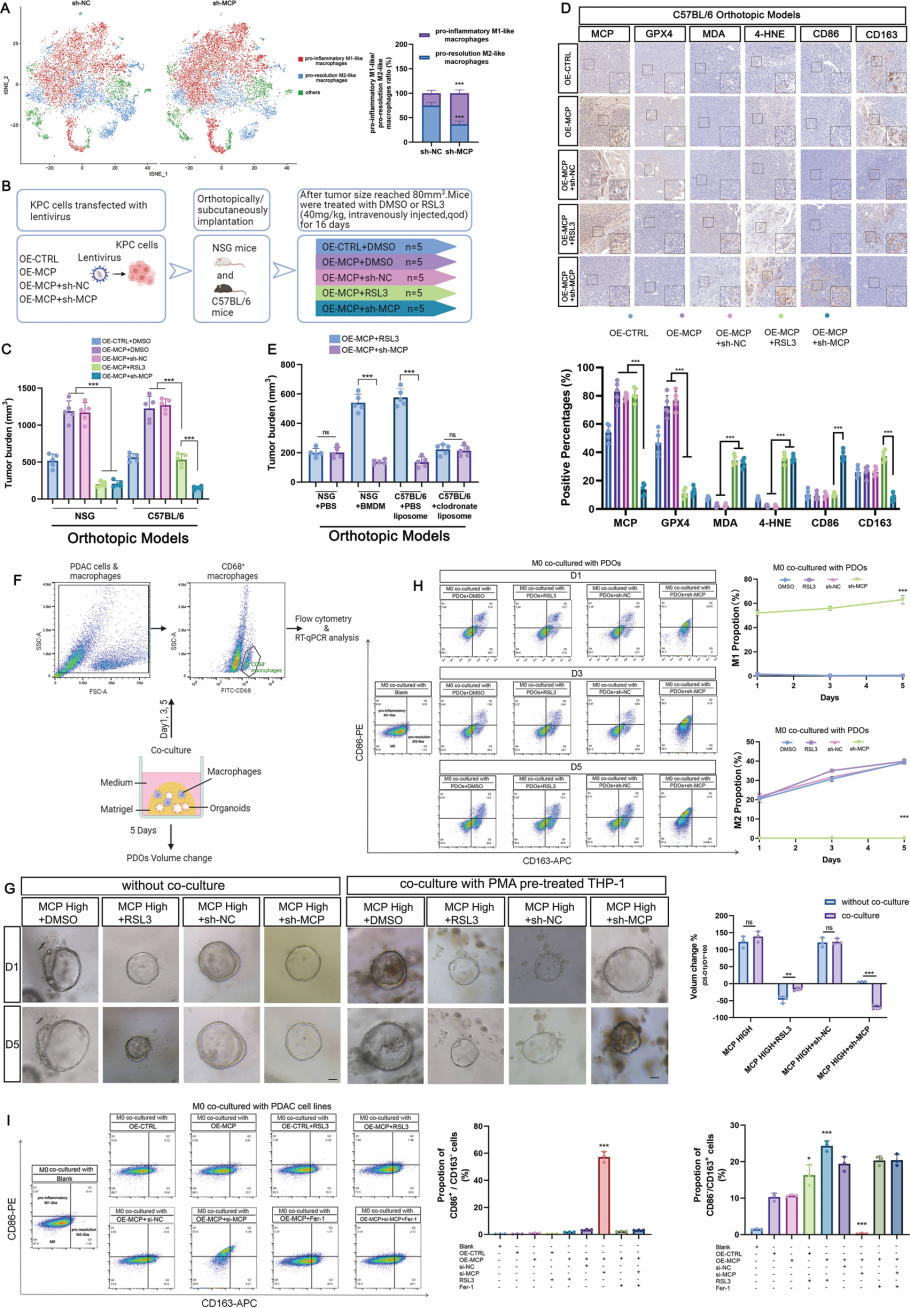
Inhibition of MCP triggered immunogenic ferroptosis to suppress pancreatic cancer by stimulating the polarization of pro‐inflammatory M1‐like macrophages. A) Single‐cell analysis of orthotopic tumor models in C57BL/6 mice. *n* = 3 for sh‐NC groups, *n* = 3 for sh‐MCP groups. B) Procedure for the transfer of tumor cells from KPC mice into NSG and C57BL/6 mice, DMSO or RSL3 (40 mg kg^−1^) was intravenously injected qod. C) Tumor growth in orthotopic models of NSG (*n*  =  5) and C57BL/6 mice (*n*  =  5). D) Immunohistochemistry analysis and quantification of the indicated markers in orthotopic models from C57BL/6 mice (*n* = 5), scale bar: 100 µm. E) Tumor growth in NSG mice co‐inoculated with BMDM and tumor cells (*n*  =  5) and in clodronate liposome‐treated C57BL/6 mice (*n*  =  5). F) Procedure for the co‐culture of PDOs with THP‐1 derived M0 macrophages. G) Representative images and statistical analysis of PDAC PDO models. After treatment with RSL3 (2 µm) or after transfection with sh‐MCP for 48 h, the PDOs were co‐cultured without or with macrophages for 5 days (*n*  =  3), scale bar: 200 µm. H) Percentages of pro‐inflammatory M1‐like and pro‐resolution M2‐like phenotypes in macrophages that were co‐cultured with the indicated PDO models for 1, 3, and 5 d (flow cytometry) (*n*  =  3). I) Percentages of pro‐inflammatory M1‐like and pro‐resolution M2‐like phenotypes in macrophages co‐cultured with OE‐CTRL or OE‐MCP‐PaTu8988t cell lines that were pre‐treated with RSL3 (2 µM) or transfected with si‐MCP for 48 h (flow cytometry) (*n*  =  3). *n* indicates the number of biological replicates (H–I) or individual mice (A–E) or PDOs (G). Data are represented as the mean ± SEM. P values are presented as ns *P* > 0.05, **P < 0.01, and ***P < 0.001. Unpaired two‐tailed Student's t‐test (A, E, G), one‐way ANOVA (C–D, I), and two‐way ANOVA (H) were used.

Besides the effects of MCP on ferroptosis, we also investigated the crosstalk between MCP and the immune microenvironment. The results from the ssGSEA, TIMER, and TISCH databases showed that MCP was closely related to macrophages. MCP level was positively associated with pro‐resolution M2‐like macrophages but negatively with pro‐inflammatory M1‐like macrophages (Figure S3A–C, Supporting Information). Next, we constructed a sh‐*MCP* orthotopic tumor model and a control model in C57BL/6 mice. Single‐cell analysis revealed increased infiltration of pro‐inflammatory M1‐like macrophages in the sh‐*MCP* group (**Figure** **3A**).

We determined the effect of MCP inhibition on PDAC in vivo. After establishing tumorigenesis in the KPC (*LSL*‐*Kras^G12D^* ^/+^ *LSL‐Trp53^R172H/+^ Pdx‐1‐Cre*) mouse model, pancreatic cancer cells from these tumors were extracted and transfected with OE‐*CTRL* or OE‐*MCP*. Mice in the OE‐*MCP* group were further transfected with sh‐negative control (*NC)* or sh‐*MCP* (Figure 3B), and the MCP protein and ferroptosis levels in the different groups were measured (Figure S3D–H, Supporting Information). We observed that MCP upregulation prevented ferroptosis, while its knockdown stimulated ferroptosis (Figure S3E–H, Supporting Information). Orthotopic and subcutaneous transplantation models were constructed by orthotopically or subcutaneously injecting KPC cells, respectively, into Rag2^−/−^ IL2rg^−/−^ double‐knockout (NSG) and C57BL/6 mice, after which dimethyl sulfoxide (DMSO) or RSL3 was administered (Figure 3B). In immunodeficient NSG mice, OE‐*MCP* promoted tumor growth, whereas sh‐*MCP* and RSL3 significantly inhibited the tumor‐promoting effect of OE‐*MCP* (Figure 3C; Figure S3I, Supporting Information). Notably, the effect on tumor suppression was significantly enhanced in C57BL/6 mice of the sh‐*MCP* group compared with that in the RSL3 group (Figure 3C; Figure S3I, Supporting Information).

Considering that MCP deficiency was accompanied by increased infiltration of the pro‐inflammatory M1‐like macrophages, we hypothesized that the macrophages were responsible for the differences in tumor suppression efficiency of sh‐*MCP* and RSL3 in C57BL/6 mice. To assess the extent of ferroptosis and the presence of macrophages, we analyzed several indicators of ferroptosis and markers specific to pro‐inflammatory M1‐like/pro‐resolution M2‐like macrophages using IHC of the tumors from C57BL/6 mice (Figure 3D; Figure S3J, Supporting Information). As expected, the expression of the ferroptosis indicators (GPX4, 4‐hydroxynonenal or 4‐HNE, and MDA) was low when MCP was expressed highly but high when RSL3 or sh‐*MCP* was administered. Importantly, we found that pro‐resolution M2‐like macrophages were the major type of macrophages in the tumor microenvironment, regardless of RSL3 administration, whereas pro‐inflammatory M1‐like macrophages were prevalent in the sh‐*MCP* groups (Figure 3D; Figure S3J, Supporting Information).

To confirm that macrophages were essential for sh‐*MCP*‐mediated tumor suppression, we co‐inoculated NSG mice with bone marrow‐derived macrophages (BMDMs) from C57BL/6 mice and KPC cells. We observed that the effectiveness of tumor inhibition was enhanced in the sh‐*MCP* group but reduced in the RSL3 group (Figure 3E; Figure S3K, Supporting Information). We also administered intravenous injections of clodronate liposomes to C57BL/6 mice to study the effect of the systemic depletion of macrophages (Figure S3L, Supporting Information). The antitumor effect was attenuated in the sh‐*MCP* group but was remarkably increased in the RSL3 group (Figure 3E; Figure S3K, Supporting Information). In addition, we collected tumors that expressed high levels of MCP from patients with PDAC to construct PDO models and treated them with RSL3 (2 µM) or sh‐*MCP*. After treatment for 48 h, we co‐cultured one aliquot of each PDO with phorbol ester (PMA)‐stimulated THP‐1 cells (Figure 3F). As macrophages differentiated from PMA‐stimulated THP‐1 cells are highly differentiated terminal cells that cannot be passaged and proliferated for a long time, we co‐cultured the macrophages and organoids for 5 days. In agreement with the results of the in vivo experiments, the antitumor effect of RSL3 in PDOs weakened when the PDOs were co‐cultured with macrophages, whereas MCP inhibition resulted in a better tumoricidal effect (Figure 3G). Macrophages were also collected from the co‐culture system on days 1, 3, and 5 for polarization analysis (Figure 3H). Macrophages with CD86^−^/CD163^+^ in flow cytometry^[^ ^32^, ^33^, ^34^ ^]^ and high expression of CD163, CD206, and Arg‐1 in RT‐qPCR were defined as pro‐resolution M2‐like macrophages, macrophages with CD86^+^/CD163^−^ in flow cytometry^[^ ^33^, ^34^, ^35^ ^]^ and high expression of CD80, CD86, and iNOS^[^ ^35^, ^36^, ^37^, ^38^ ^]^ in RT‐qPCR were defined as pro‐inflammatory M1‐like macrophages. The results of flow cytometry and RT‐qPCR showed that the macrophages polarized toward the pro‐resolution M2‐like phenotype during co‐culture with PDOs, irrespective of RSL3 treatment (Figure 3H; Figure S3M, Supporting Information). However, pro‐inflammatory M1‐like polarization of macrophages was stimulated in PDOs with MCP knockdown (Figure 3H; Figure S3M, Supporting Information). The results from the in vivo experiments and PDOs demonstrated that MCP inhibition effectively suppressed PDAC by stimulating both ferroptosis and polarization of pro‐inflammatory M1‐like macrophages.

Considering the potential impact of ferroptotic cancer cells on the immune microenvironment, we investigated whether ferroptosis, triggered by MCP deficiency, influenced pro‐inflammatory M1‐like macrophage polarization. Toward this, we co‐cultured ferroptotic PDAC cells, stimulated with RSL3 or si‐*MCP*, with macrophages derived from PMA‐treated THP‐1 cells. After 24 h, the macrophages were collected for further analysis. The results of flow cytometry and RT‐qPCR showed an increase in the proportion of the pro‐resolution M2‐like macrophages when the macrophages were co‐cultured with the OE‐*CTRL*, OE‐*MCP*, and RSL3‐stimulated ferroptotic cells (Figure 3I; Figure S3N, Supporting Information). Conversely, macrophages co‐cultured with the si‐*MCP*‐triggered ferroptotic cells showed a significantly high proportion of the pro‐inflammatory M1‐like phenotype (Figure 3**I**; Figure S3N, Supporting Information). However, administration of ferroptosis inhibitors in the si‐*MCP* group eliminated polarization toward the pro‐inflammatory M1‐like phenotype and led to the predominance of the pro‐resolution M2‐like macrophages (Figure 3I; Figure S3N, Supporting Information), indicating that pro‐inflammatory M1‐like polarization was stimulated by si‐*MCP*‐induced ferroptosis. Altogether, these results demonstrated that ferroptosis triggered by MCP inhibition can stimulate tumoricidal pro‐inflammatory M1‐like macrophages to enhance the efficiency of tumor suppression.

### 2.4 Lipid Nanoparticles Targeting MCP Demonstrated Promising Tumor Suppression in Pancreatic Cancer

**Figure 4:**
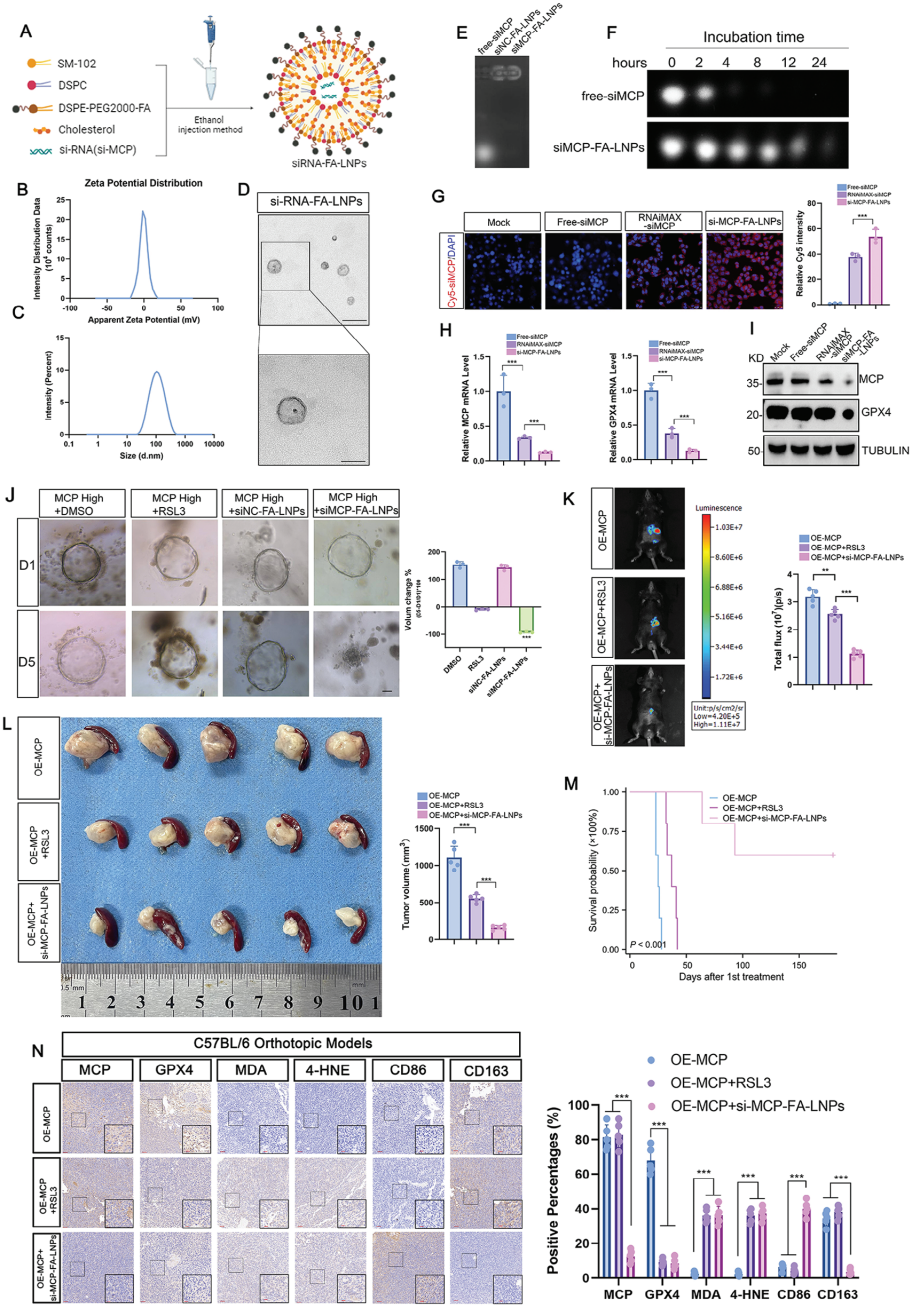
Lipid nanoparticles (LNPs) targeting MCP demonstrated promising tumor suppression in pancreatic cancer. A) Methods used to construct si*MCP‐*FA‐LNPs. B) Zeta potential distribution of si*MCP‐*FA‐LNPs. C) Size distribution of si*MCP‐*FA‐LNPs as determined by DLS. D) Electron microscopy of si*MCP‐*FA‐LNPs, scale bar: 200 nm [top] and 100 nm [bottom]. E) Electrophoretic mobility of free si*MCP*, si*NC*‐FA‐LNPs, and si*MCP‐*FA‐LNPs. F) The serum stability of free si*MCP* and si*MCP*‐FA‐LNPs was visualized by agarose gel electrophoresis at different incubation durations. G) Representative images and statistical analysis of Cy5‐si*MCP* in PANC‐1 cells after the indicated treatments (*n*  =  3), scale bar: 25 µm. H,I) The suppression efficiency of MCP and GPX4 in the indicated groups at the mRNA (H) and protein (I) level (*n*  =  3). J) Representative images and statistical analysis of PDAC PDO models. After treatment with RSL3 (2 µm) or si*MCP‐*FA‐LNPs (si*MCP* 50nm) for 48 h, the PDOs were co‐cultured with macrophages for 5 days (*n* = 3), scale bar: 200 µm. K) Representative images and statistical analysis of in vivo bioluminescence in orthotopic tumor models with the indicated treatments (*n* = 5). L) Tumors were harvested from the mice 16 days after the first treatment, tumor volume were measured (*n*  =  5). M) Overall survival time of C57BL/6 mice with the indicated treatments (*n*  =  5). N) Representative images and statistical analysis of immunohistochemistry in C57BL/6 mice. Scale bar: 100 µm (*n*  =  5). n indicates the number of biological replicates (G–H) or individual mice (K–N) or PDOs (J). Data are represented as the mean ± SEM. P values are presented as ns *P* > 0.05, **P < 0.01, and ***P < 0.001. Log‐rank test (M) and one‐way ANOVA (G–H, J–L, and N) were used.

As MCP inhibition showed remarkable tumor eradication efficiency in our in vivo and PDO‐based experiments, we speculated that targeting MCP could be a promising therapy for PDAC. Therefore, we constructed lipid nanoparticles (LNPs) composed of DSPE‐polyethylene glycol (2000)‐folate (FA), DSPC, cholesterol, and SM‐102 for delivering si‐*MCP* (**Figure** **4**A,B). Using dynamic light scattering, the diameters of siRNA‐FA‐LNPs were measured to be 131.2 nm (PDI: 0.054) (Figure 4C), which was further confirmed using transmission electron microscopy (Figure 4D). The encapsulation efficiency of the LNPs was 96% in the RiboGreen assay. The agarose gel electrophoretic shift assay did not reveal any detectable shift in the siRNA‐FA‐LNPs compared to that of the free siRNA, confirming the effective packaging ability of the LNPs (Figure 4E). Moreover, the LNPs significantly improved the stability of the siRNA in serum (Figure 4F). By incubating the free‐Cy5‐si*MCP*, RNAiMAX‐Cy5‐siMCP, or Cy5‐ siMCP‐LNPs with PANC‐1 cells, our LNPs demonstrated the effectively si*MCP* delivery ability as the PDAC cells treated by LNPs showed the highest Cy5 fluorescence intensity (Figure 4G), subsequently the LNPs efficiently inhibited MCP and GPX4 (Figure 4H,I).

Expression of the folate receptor is elevated in many cancers, rendering it a valuable target for tumor‐specific drug delivery.^[39]^ According to the GEPIA2 database, PDAC also demonstrated significant upregulation of folate receptor alpha (FOLR1) (Figure S4A, Supporting Information). Consequently, we used FA‐conjugated LNPs to target PDAC and verified the specific tumor distribution of Cy5‐siRNA‐LNPs in tumor‐bearing C57BL/6 mice using fluorescence imaging (Figure S4B, Supporting Information). Weight changes and hematoxylin‐eosin staining in mice confirmed the general safety of these LNPs (Figure S4C,D, Supporting Information).

We examined the tumor‐suppressive effects of these LNPs on pancreatic cancer cells. First, in the PDO models with macrophages, si*MCP‐*FA‐LNPs showed better therapeutic effect than saline, RSL3, or si*NC‐*FA‐LNPs (Figure 4J). In the orthotopic and subcutaneous transplantation models (Figure S4E, Supporting Information), si*MCP*‐FA‐LNPs showed stronger tumoricidal effect than RSL3 (Figure 4K–M; Figure S4G,H, Supporting Information), as they effectively suppressed MCP (Figure S4F, Supporting Information) and significantly inhibited tumor growth (Figure 4K–L; Figure S4G, Supporting Information), prolonging mouse survival (Figure 4M; Figure S4H, Supporting Information). IHC analysis confirmed that the levels of ferroptosis and percentage of pro‐inflammatory M1‐like macrophages were effectively increased in the si*MCP‐*FA‐LNPs‐treated groups (Figure 4N; Figure S4I, Supporting Information). Collectively, the si*MCP‐*loaded LNPs demonstrated superior antitumor efficiency in multiple PDAC models.

### 2.5 Inhibition of MCP Induced Pro‐Inflammatory M1‐Like Macrophage Polarization by Stimulating Nucleocytoplasmic Transportation and Early Release of HMGB1

**Figure 5:**
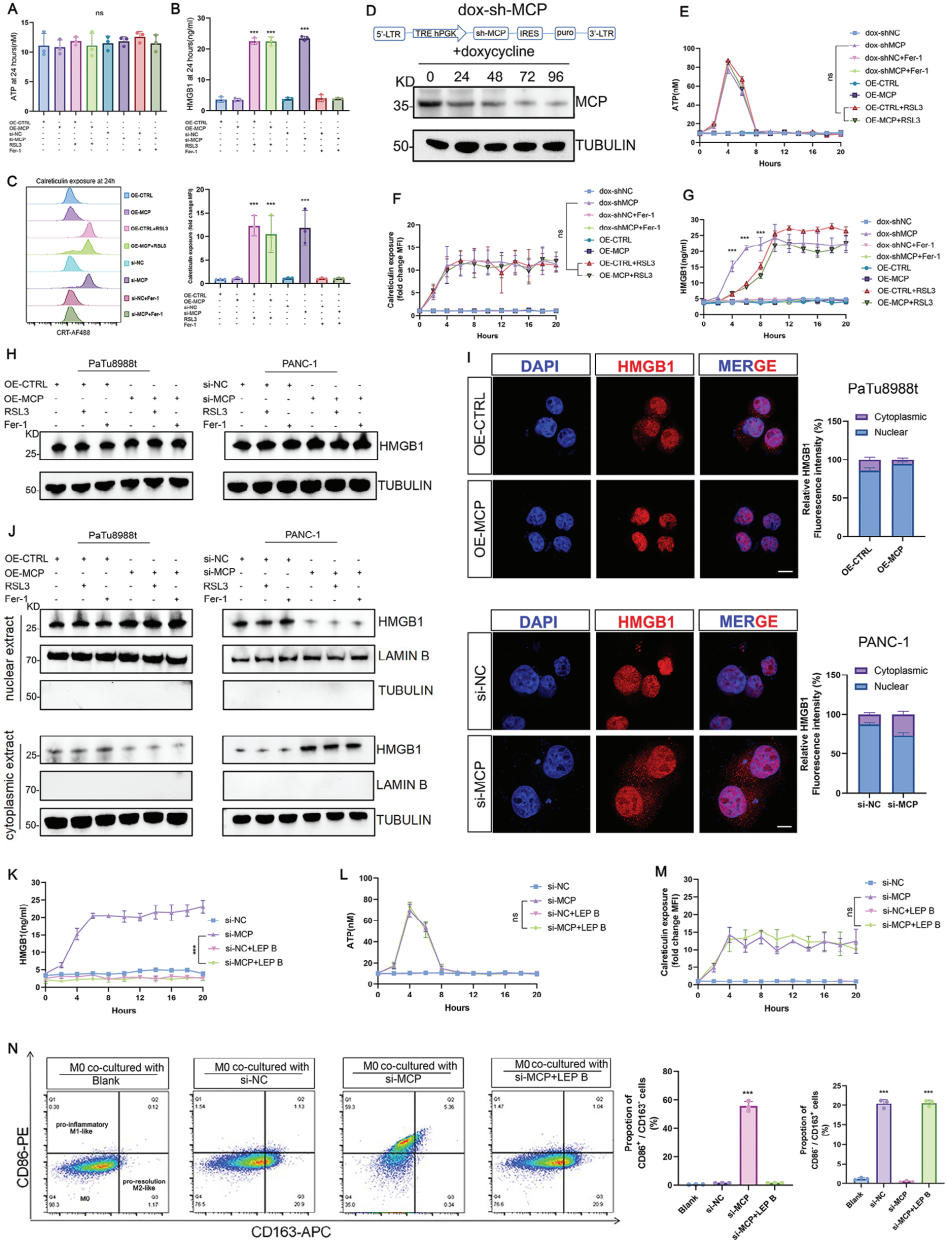
Inhibition of MCP induced pro‐inflammatory M1‐like macrophage polarization by stimulating nucleocytoplasmic transport and early release of HMGB1. A,B) ELISA of ATP (A) and HMGB1 B) levels in the supernatants of control, ferroptosis‐resistant, and ferroptotic groups in PANC‐1 cells (*n*  =  3). C) Analysis of calreticulin exposure on the PANC‐1 cell surface in different groups, histograms represent the mean fold change in fluorescence intensity generated by comparing the mean fluorescence intensity (MFI) of treated cells to that of OE‐CTRL cells (*n*  =  3). D) MPC protein level in sh‐MCP PANC‐1 cells after doxycycline treatment. E) Temporal changes in ATP levels in the supernatant of PANC‐1 cells following the indicated treatments (*n*  =  3). F) Temporal changes in calreticulin exposure on the surface of PANC‐1 cells after the indicated treatments (*n*  =  3). G) Temporal changes in HMGB1 levels in the supernatant of PANC‐1 cells following the indicated treatments (*n*  =  3). H) Total protein levels of HMGB1 in PDAC cells after the indicated treatments (*n*  =  3). I) Representative images and statistical analysis of immunofluorescence of HMGB1 in PaTu8988t and PANC‐1 cells (*n* = 3), scale bar: 10 µm. J) Nuclear and cytoplasmic levels of HMGB1 in PDAC cells after the indicated treatments. K–M, Temporal changes in HMGB1 K), ATP L), and calreticulin exposure M) levels in the supernatant of PANC‐1 cells after treatment with leptomycin B (*n*  =  3). N) Percentages of pro‐inflammatory M1‐like and pro‐resolution M2‐like phenotypes in macrophages co‐cultured with leptomycin B‐treated si‐MCP PANC‐1 cells (flow cytometry) (*n*  =  3). n indicates the number of biological replicates. Data are represented as the mean ± SEM. *P* values are presented as ns *P* > 0.05, **P < 0.01, and ***P < 0.001. Unpaired two‐tailed Student's *t*‐tests (I), one‐way ANOVA (A–C, and N), and two‐way (E–G, K–M) ANOVA were used.

To investigate the mechanism underlying the polarization of pro‐inflammatory M1‐like macrophages induced by MCP‐dependent ferroptosis, we constructed the control groups (OE‐*CTRL* and si‐*NC*), ferroptosis‐resistant groups [OE‐*MCP*, si‐*NC* treated with Fer‐1 (1 µM), and si‐*MCP* treated with Fer‐1 (1 µM)], and ferroptotic groups [OE‐*CTRL* treated with RSL3 (2 µM), OE‐*MCP* treated with RSL3 (2 µM), and si‐*MCP*] in PANC‐1 cell lines. The cells and supernatants from the above groups were harvested 24 h after treatment to identify the factors that activated the polarization of pro‐inflammatory M1‐like macrophages [interferon (IFN)‐ γ, tumor necrosis factor (TNF)‐α,^[^ ^40^, ^41^ ^]^ and DAMPs, such as HMGB1, ATP, and CRT.^[^ ^14^, ^17^, ^18^, ^19^ ^]^] The results from enzyme‐linked immunosorbent assay (ELISA) revealed that the IFN‐γ and TNF‐α levels in the supernatants did not change remarkably (Figure S5A,B, Supporting Information). Regarding the DAMPs, ATP levels remained unchanged, but the levels of HMGB1 in the supernatants and CRT‐exposed cells were considerably high in all ferroptotic groups (**Figure** **5**A–C). Nevertheless, these findings did not elucidate the mechanism underlying the induction of ferroptosis by si‐*MCP*, which promoted the polarization of the pro‐inflammatory M1‐like macrophages.

As the amount and kinetics of DAMP release may influence immunogenicity, we constructed doxycycline‐induced sh‐*MCP* PANC‐1 cell lines (dox‐sh*MCP*) to better determine the extent and timing of DAMP release. We noticed that MCP protein expression in dox‐sh*MCP* cells was significantly suppressed 72 h after doxycycline treatment (Figure 5D). Subsequently, following the administration of doxycycline for 72 h, we analyzed dox‐sh*MCP* cells and their supernatants over 20 h to determine the pattern of DAMP release. We found that the release patterns of IFN‐γ, TNF‐α, and ATP and exposure to CRT did not differ among the ferroptotic groups induced by RSL3 or dox‐sh*MCP* (Figure 5E,F; Figure S5C,D, Supporting Information). ATP was released within 3–6 h after the stimulation of ferroptosis (Figure 5E), and CRT exposure was detected 2 h after induction of ferroptosis (Figure 5F). Furthermore, the levels of IFN‐γ and TNF‐α in the supernatant of the ferroptotic cells did not change (Figure S5C,D, Supporting Information). However, the release of HMGB1 in dox‐sh*MCP*‐induced ferroptotic cells occurred significantly earlier than that in RSL3‐induced ferroptotic cells (Figure 5G). In particular, increase in HMGB1 levels was not observed until 8 h after RSL3 administration. In contrast, HMGB1 level was significantly elevated in the dox‐sh*MCP* group during the initial 3–6 h, although this elevation was suppressed by a ferroptosis inhibitor (Figure 5G). Collectively, ferroptotic cells induced by RSL3 or sh‐*MCP* released adjuvants with potential immunogenic activities, such as ATP and HMGB1, or showed CRT exposure. However, among these adjuvants, only the release pattern of HMGB1 differed significantly between the RSL3 and sh‐*MCP* groups.

As the amount and kinetics of HMGB1 release were altered in sh*MCP*‐induced ferroptotic cells, we determined whether MCP could regulate the expression or subcellular localization of HMGB1. We did not detect any changes in the mRNA and protein levels of HMGB1 among the control, ferroptosis‐resistant, and ferroptotic groups (Figure 5H; Figure S5E, Supporting Information), however, immunofluorescence revealed increased cytosolic accumulation of HMGB1 in si‐*MCP* cells but decreased cytosolic localization of HMGB1 in OE‐*MCP* cells (Figure 5I). To verify whether si‐*MCP* promoted the cytoplasmic transport of HMGB1, we assessed the nuclear and cytoplasmic fractions for the presence of HMGB1. Cytosolic HMGB1 level increased in the si‐*MCP* cells but decreased in the OE‐*MCP* cells. In addition, RSL3 or Fer‐1 administration did not affect the nucleocytoplasmic transportation of HMGB1 (Figure 5J). To investigate whether the cytoplasmic translocation of HMGB1 promoted its early release, we used leptomycin B (LEP‐B, a nuclear export inhibitor, 50 ng/mL, 12 h) to restrict the cytoplasmic shift of HMGB1 (Figure S5F, Supporting Information). The results showed that LEP‐B did not affect the exposure of CRT or the release pattern of ATP, but prevented the early release of HMGB1, and reduced the number of pro‐inflammatory M1‐like macrophages in the si‐*MCP* treated groups (Figure 5K–N; Figure S5G, Supporting Information). Besides, LEP‐B did not affect si‐*MCP*‐induced ferroptosis (Figure S5H–K, Supporting Information). These results indicated that the augmented cytoplasmic translocation of HMGB1 occurred only when MCP was blocked and not during the administration of RSL3. Following the initiation of ferroptosis triggered by MCP inhibition, the accumulation of cytosolic HMGB1 facilitated its early release, which was essential for pro‐inflammatory M1‐like macrophage polarization.

### 2.6 MCP Mediated the Nucleocytoplasmic Transportation of HMGB1 by Modulating the UFMylation of PIR

**Figure 6:**
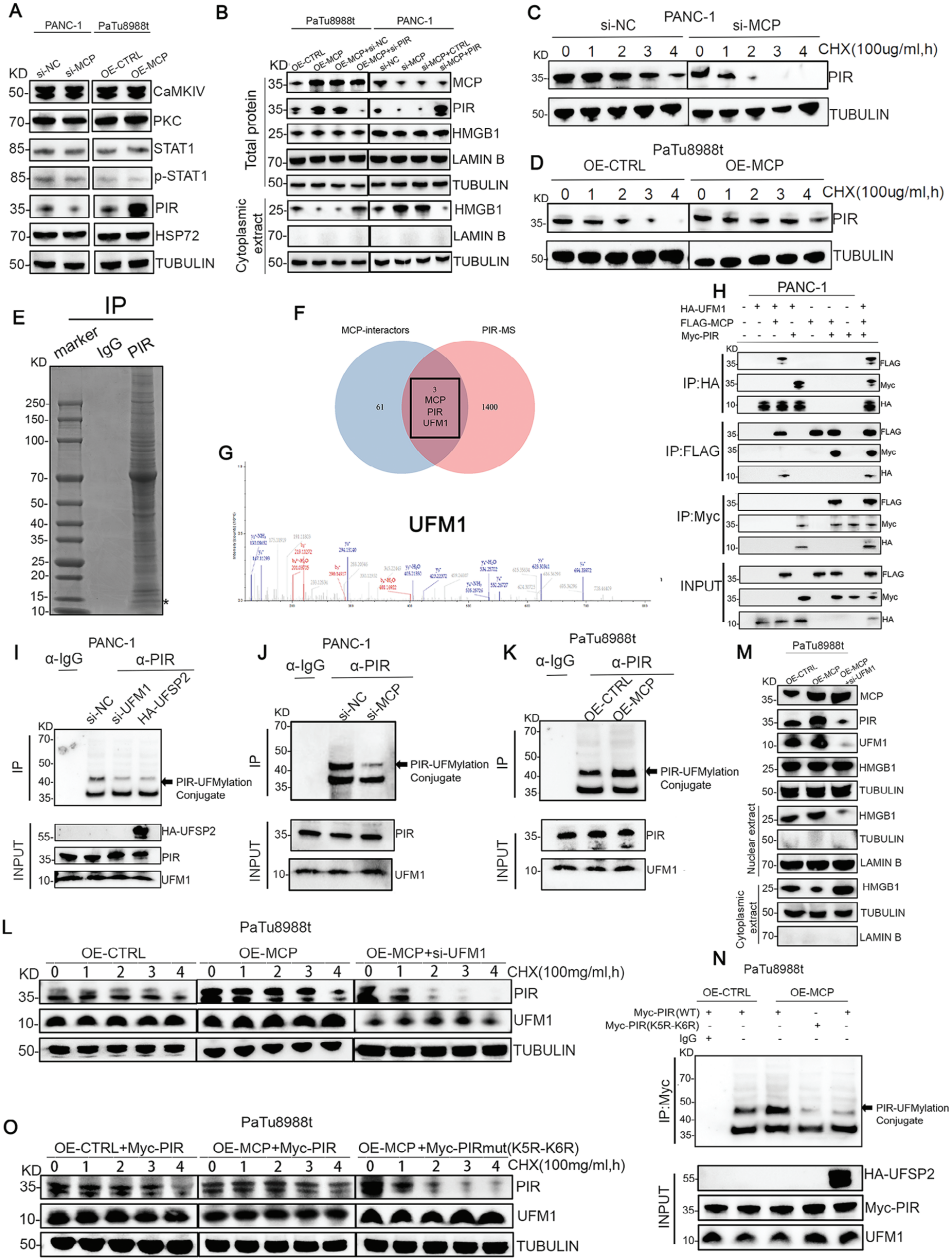
MCP mediated the nucleocytoplasmic transport of HMGB1 by modulating the UFMylation of PIR. A) CaMK4, PKC, STAT1, PIR, and HSP72 protein levels in the OE‐*MCP* and si‐*MCP* groups. B) Protein levels of MCP, PIR, and HMGB1 in the OE‐*MCP* and si‐*MCP* groups transfected with si‐*PIR* and *PIR*, respectively. C,D) Temporal changes in PIR protein level in si‐*MCP* (C) and OE‐*MCP* (D) groups after cycloheximide treatment (CHX, 100 µg mL^−1^). E) PANC‐1 cell lysates were immunoprecipitated using control IgG or anti‐*PIR* antibody, followed by Coomassie Blue staining. F) Venn diagram based on the results of mass spectrometry (Figure 6E) and MCP‐interacting proteins according to BioGRID (thebiogrid. org/). G) Best unique peptide spectrum match of UFM1. H) Interaction between MCP, PIR, and UFM1 was analyzed using co‐immunoprecipitation. I) Co‐immunoprecipitation analysis of PIR UFMylation in the si‐*UFM1* and HA‐*UFSP2* groups. J,K) Co‐immunoprecipitation analysis of PIR UFMylation in si‐*MCP* (J) and OE‐*MCP* (K) cells. L) Changes in PIR and UFM1 protein levels in OE‐*MCP*‐PaTu8988t cells transfected with si‐*UFM1*. M) Protein levels of MCP, PIR, UFM1, and HMGB1 in OE‐*MCP*‐PaTu8988t cells transfected with si‐*UFM1*. N) PIR UFMylation in OE‐*MCP* cells transfected with Myc‐PIR (wild type, WT) or Myc‐PIR (with K5‐K6 lysines mutated to arginine). O) Temporal changes in protein levels of PIR in OE‐*MCP* cells transfected with Myc‐PIR (wild type, WT) or Myc‐PIR (mutation type, mut, with K5‐K6 lysines mutated to arginines).

Several regulators are closely associated with the subcellular localization of HMGB1, such as PKC,^[42]^ CaMK4,^[43]^ STAT1,^[44]^ HSP72,^[45]^ and PIR.^[46]^ Among these, we found that PIR protein level was upregulated in OE‐*MCP* cells but downregulated in si‐*MCP* cells (**Figure** **6**A), however, the mRNA levels of all these regulators remained unchanged in both OE‐*MCP* and si‐*MCP* groups (Figure S6A, Supporting Information). The protein‐protein association networks of MCP in the STRING database (https://string‐db.org) also showed a potential relationship between MCP and PIR (Figure S6B, Supporting Information). In addition, si‐*PIR* increased the cytoplasmic translocation of HMGB1 in OE‐*MCP* cells. OE‐*PIR* decreased the cytoplasmic protein levels of HMGB1 in si‐*MCP* cells (Figure 6B) but did not affect the total protein or mRNA levels of HMGB1 (Figure 6B; Figure S6C, Supporting Information). These results indicated that MCP can manipulate the cytoplasmic translocation of HMGB1 via PIR.

To better understand how MCP regulates PIR levels, we blocked protein synthesis using cycloheximide (CHX) and assessed PIR degradation in the OE‐*MCP* and si‐*MCP* groups. The findings revealed that si‐*MCP* accelerated the decomposition of PIR (Figure 6C; Figure S6D, Supporting Information), whereas the opposite effect was observed in the OE‐*MCP* group (Figure 6D; Figure S6E, Supporting Information). To investigate how MCP regulated PIR degradation, we collected PIR and their associated proteins from PDAC cell lysates using protein A/G agarose beads conjugated with anti‐PIR antibodies (Figure 6E). Based on the results of mass spectrometry (MS), the PIR‐associated proteins were intersected with the MCP‐associated proteins from BioGRID (thebiogrid.org/), and identified UFM1 (mediates UFMylation of target proteins and increases their stability and function^[^ ^47^, ^48^, ^49^ ^]^) might potentially interact with MCP and PIR (Figure 6F,G). Simulated molecular docking also indicated that UFM1 interacted with PIR (Figure S6F, Supporting Information). In addition, co‐immunoprecipitation using exogenously tagged proteins confirmed interactions between MCP, PIR, and UFM1 (Figure 6H). Based on these findings, we hypothesized that PIR is a substrate of UFM1 and that MCP regulates PIR level by regulating its UFMylation.

Following the suppression of UFM1 or the induction of UFSP2, an enzyme that specifically reverses the connection between UFM1 and target proteins,^[50]^ we performed a UFMylation assay and verified that PIR was a substrate of UFM1, as UFMylation of PIR was suppressed specifically (Figure 6I). Additionally, we confirmed that PIR UFMylation was regulated by MCP, as it decreased in si‐*MCP* cells (Figure 6J) but increased in OE‐*MCP* cells (Figure 6K). To confirm whether MCP‐enhanced UFMylation was responsible for the elevation in PIR protein levels, we transfected si‐*UFM1* into OE‐*MCP* cells. si‐UFM1 reversed the effects of OE‐*MCP*, leading to accelerated degradation of (Figure 6L; Figure S6G, Supporting Information) and reduction in PIR protein level (Figure 6M), ultimately promoting the cytoplasmic translocation of HMGB1 (Figure 6M), but not affected the mRNA level of HMGB1 (Figure S6H, Supporting Information). UFM1 can recognize a motif containing the amino acid sequence KKVT,^[^ ^48^, ^49^ ^]^ and we consistently identified a KKVT sequence in PIR (Figure S6I, Supporting Information). To determine whether these lysines were the preferred UFMylation sites, we mutated the K5 and K6 lysines in KKVT to arginines (K5R and K6R). The results demonstrated that these mutations strongly decreased UFMylation (Figure 6N) and accelerated the degradation of PIR in the OE‐*MCP* group (Figure 6O **;** Figure S6J, Supporting Information), indicating that the K5 and K6 lysines were the UFMylated sites in PIR. Altogether, the above results showed that MCP modulated the nucleocytoplasmic transport of HMGB1 by altering the UFMylation of PIR.

### 2.7 PIR Regulated the MCP‐Mediated Change in GPX4 Transcription

**Figure 7:**
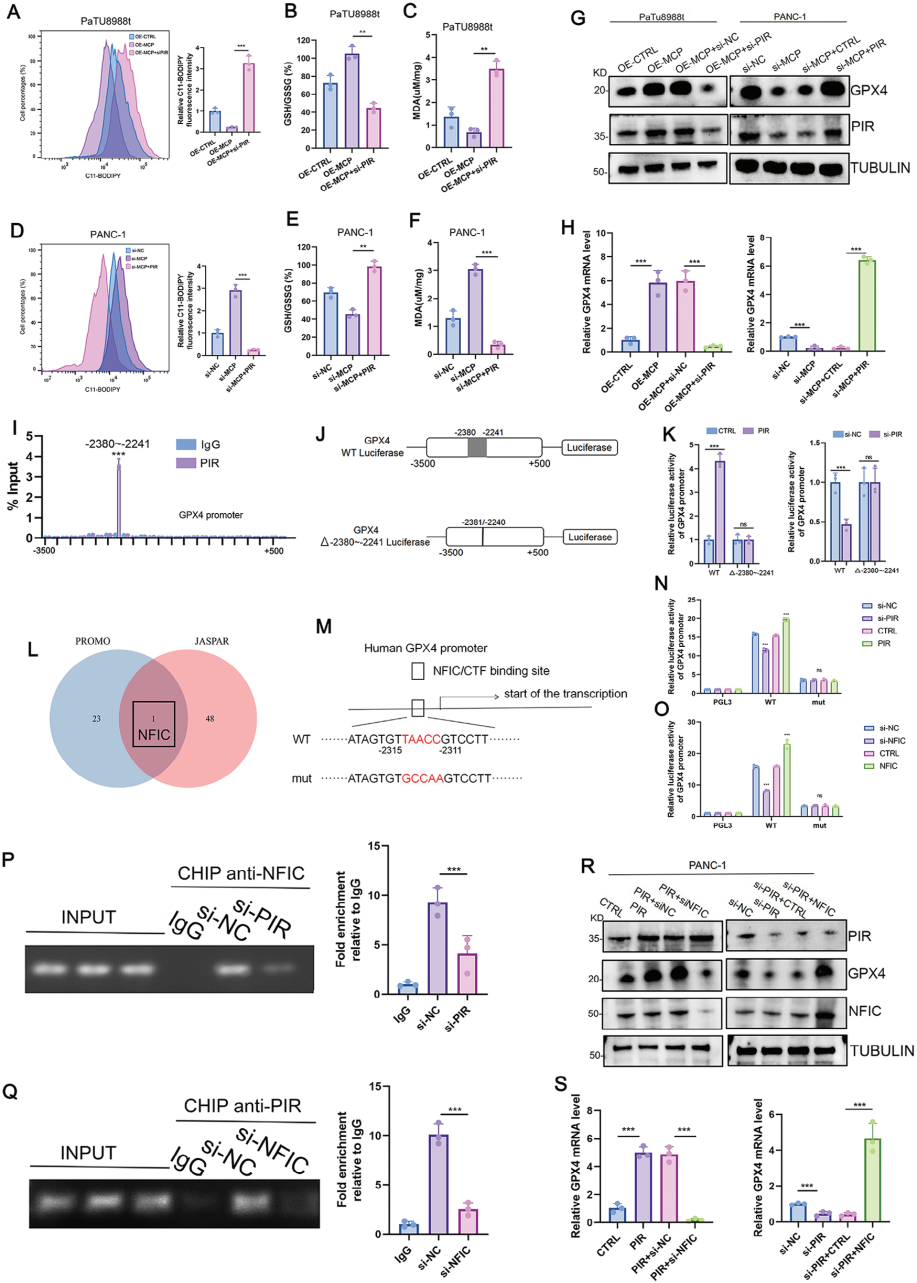
PIR regulated the MCP‐mediated change in GPX4 transcription. A) Levels of lipid peroxidation in OE‐*MCP*‐PaTu8988t cells transfected with si‐*PIR* measured by flow cytometry, fluorescence intensity was normalized to the mean value of three replicates of the OE‐*CTRL* groups (*n* = 3). B,C) Levels of GSH/GSSG ratio (B) and MDA (C) in OE‐*MCP*‐PaTu8988t cells transfected with si‐*PIR* (*n* = 3). D) Levels of lipid peroxidation in si‐*MCP*‐PANC‐1 cells transfected with *PIR* measured by flow cytometry, fluorescence intensity was normalized to the mean value of three replicates of the si‐*NC* groups (*n* = 3). E,F) Levels of GSH/GSSG ratio (E) and MDA (F) in si‐*MCP*‐PANC‐1 cells transfected with *PIR* (*n* = 3). G,H) Protein (G) and mRNA levels (H) of GPX4 in OE‐*MCP*‐PaTu8988t cells transfected with si‐*PIR* and in si‐*MCP*‐PANC‐1 cells transfected with *PIR* (*n*  =  3). I) ChIP‐qPCR analysis of the region spanning ‐3000 to +500 bp in the *GPX4* promoter. PIR antibody or IgG was used for immunoprecipitation in PANC‐1 cells. J) Schematic showing the *GPX4* promoter‐luciferase reporter constructs with wild type (WT) and the ‐2380 to ‐2241 deletion mutation. K) Luciferase fluorescence with different *GPX4* promoters in OE‐*PIR*‐PANC‐1 cells and si‐*PIR*‐PANC‐1 cells (*n*  =  3). L) Venn diagram showing transcription factors from JASPAR (jaspar.genereg.net) and PROMO (alggen.lsi.upc.es/cgi‐bin/promo_v3/promo) that bind the ‐2380 to ‐2241 region of the *GPX4* promoter. M) WT and mutant binding sites (mut) of NFIC on the human *GPX4* promoter. N, Luciferase reporter assay of different *GPX4* promoters (PGL3, WT, and mutant) in OE‐*PIR* and si‐*PIR*‐PANC‐1 cells (*n*  =  3). O) Luciferase reporter analysis of different *GPX4* promoters (PGL3, WT, and mutant) in OE‐*NFIC* and si‐*NFIC*‐PANC‐1 cells (*n*  =  3). P,Q) Chromatin immunoprecipitation showing the binding of NFIC and PIR to the promoter of *GPX4* in si‐*PIR* and si‐*NFIC*‐PANC‐1 cells, respectively (*n*  =  3). R,S) protein (R) and mRNA (S) levels of *GPX4* were determined after si‐*NFIC* and *NFIC* were introduced into OE‐*PIR* and si‐*PIR*‐PANC‐1 cells, respectively (*n*  =  3). n indicates the number of biological replicates. Data are represented as the mean ± SEM. P values are presented as ns *P* > 0.05, **P < 0.01, and ***P < 0.001. One‐way (A–F, H, P–Q, and S) and two‐way (I, K, and N–O) ANOVA were used.

In addition to HMGB1 regulation, we investigated whether PIR was involved in *MCP*‐related ferroptosis. We found that suppression of PIR elevated the levels of lipid peroxidation and MDA and reduced the GSH/GSSG ratio in OE‐*MCP* cells (**Figure** **7**A–C; Figure S7A, Supporting Information). Similarly, the levels of lipid peroxidation and MDA and the GSH/GSSG ratio, which were altered by si‐*MCP* treatment, were reversed by PIR replenishment (Figure 7D–F; Figure S7B, Supporting Information). Moreover, OE‐*PIR* increased the protein and mRNA levels of *GPX4* in the si‐*MCP* group, whereas si‐*PIR* decreased the protein and mRNA levels of *GPX4* in the OE‐*MCP* group (Figure 7G,H). Altogether, these results indicated that MCP influenced *GPX4* transcription via PIR.

To determine how PIR regulated *GPX4* transcription, we performed chromatin immunoprecipitation‐qPCR (ChIP‐qPCR) to determine the binding of PIR to the −2380 to −2241 region in the *GPX4* promoter (Figure 7I). Based on the results of the ChIP‐qPCR assay, we constructed the *GPX4* promoter‐luciferase reporter gene construct harboring the wild type or the −2380 to −2241 deleted promoter (Figure 7J). We found that neither *PIR* nor si‐*PIR* affected the expression of the luciferase reporter when the −2380 to −2241 region was deleted. However, expression of the wild‐type luciferase reporter was enhanced by *PIR* but suppressed by si‐*PIR* (Figure 7K). These results indicated that PIR bound to the promoter region of *GPX4*, thereby promoting its transcription.

As a transcriptional coactivator, PIR may regulate *GPX4* transcription in the presence of an appropriate transcription factor. Therefore, we searched the JASPAR (jaspar.genereg.net) and PROMO databases (alggen.lsi.upc.es/cgi‐bin/promo_v3/promo) using the sequence of the −2380 to −2241 region and identified the transcription factor, NFIC (Figure 7L). We then developed luciferase reporter plasmids containing the wild type (WT) or mutant (mut) predicted binding site of NFIC on the *GPX4* promoter (Figure 7M; Figure S7C, Supporting Information). Luciferase activity was enhanced when PIR or NFIC was upregulated and reduced when PIR or NFIC was suppressed (Figure 7N–O). In addition, luciferase activity was not modified by PIR and NFIC in the mutant (Figure 7N–O).

Next, we designed primers based on the predicted binding site and performed a ChIP assay (Figure S7D, Supporting Information). The results verified that both NFIC and PIR bound to the predicted binding site (Figure 7P–Q; Figure S7E,F, Supporting Information). Furthermore, the downregulation of PIR or NFIC reduced the binding of NFIC and PIR to the *GPX4* promoter, respectively (Figure 7P,Q). Conversely, the upregulation of PIR or NFIC increased the binding of NFIC and PIR, respectively, to the *GPX4* promoter (Figure S7E,F, Supporting Information). In addition, the elevation in the mRNA and protein levels of GPX4 mediated by PIR was suppressed by si‐*NFIC*, and the decrease in the same mediated by si‐*PIR* was reversed by NFIC overexpression (Figure 7R,S). These results confirmed the specific binding sites of PIR and NFIC on the *GPX4* promoter region and further indicated that PIR and NFIC act as complementary regulators of *GPX4* transcription. Collectively, these results suggested that MCP regulated *GPX4* transcription via the PIR–NFIC complex to prevent ferroptosis.

### 2.8 MCP Regulated the Levels of GPX4 and Pro‐Inflammatory M1‐Like Macrophage Infiltration in Patients with Pancreatic Cancer

**Figure 8:**
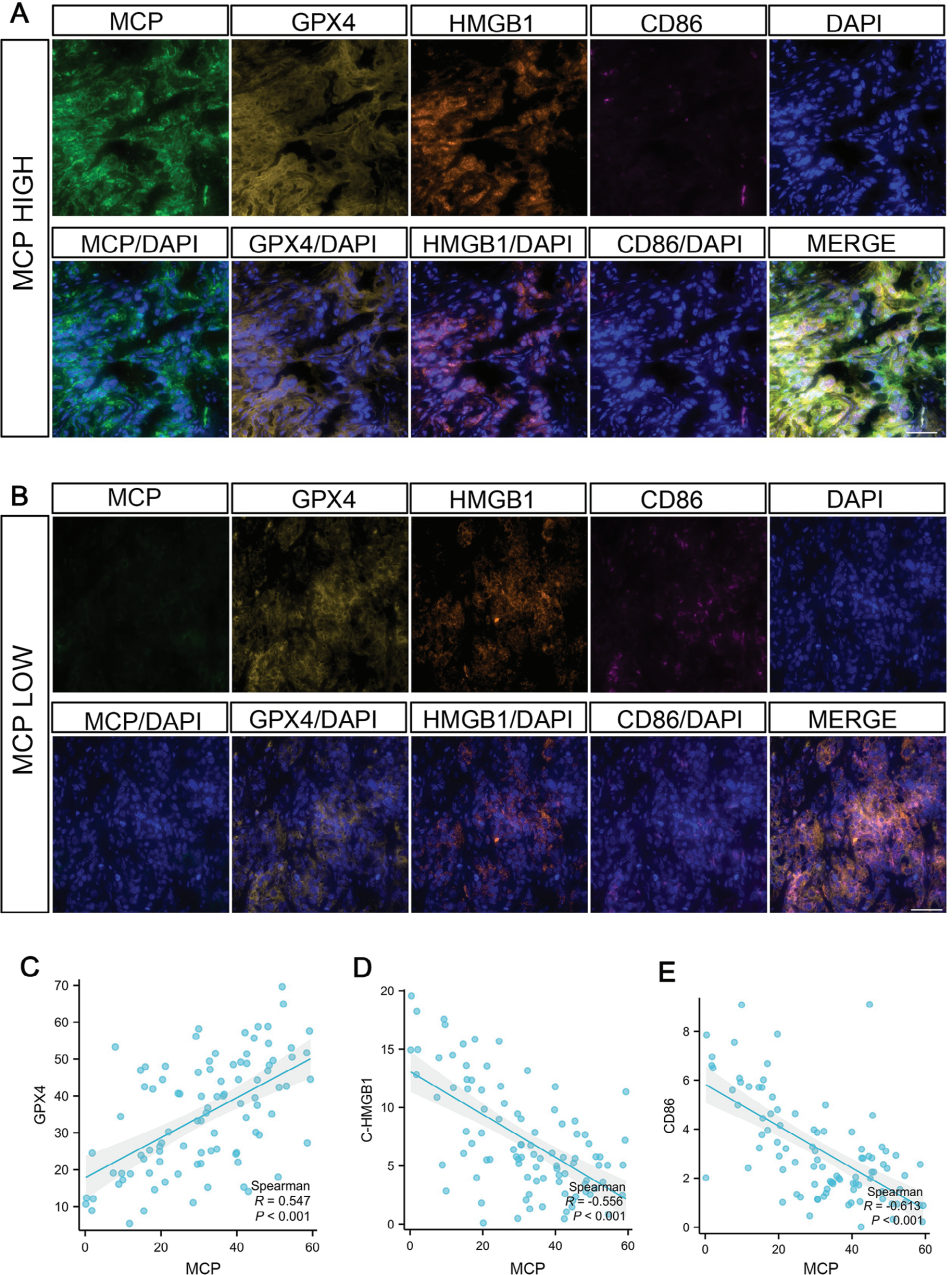
MCP regulated the levels of GPX4 and pro‐inflammatory M1‐like macrophage infiltration in patients with pancreatic cancer. A,B) Immunofluorescence analysis of specimens from representative patients with PDAC. Markers such as MCP, HMGB1, GPX4, and CD86 were assessed. Scale bar: 50 µm. C–E) Correlation between MCP and GPX4 (C), MCP and cytoplasmic HMGB1 (C‐HMGB1) (D), and MCP and CD86 (E) in patients with PDAC (*n* = 98). *n* indicates the number of individual patients. Spearman analysis (C–E) was used.

We measured the levels of MCP, GPX4, HMGB1, and CD86 in patients with PDAC to verify the connection between MCP, ferroptosis, and immunogenicity. In agreement with the above results, patients with high MCP expression harbored high GPX4 but low cytosolic HMGB1 levels, along with negligible amounts of tumoricidal pro‐inflammatory M1‐like macrophages (**Figure** **8**A,C–E). Conversely, patients with low MCP levels exhibited reduced GPX4 levels, increased cytoplasmic HMGB1 levels, and notable infiltration of pro‐inflammatory M1‐like macrophages. (Figure 8B,C–E). These results revealed that MCP was an indicator of immunogenic ferroptosis in PDAC, and that patients with high MCP expression may exhibit immunosuppressive microenvironments and resistance to ferroptosis.

## 3 Discussion

Induction of ferroptosis can be lethal for many types of cancers, including pancreatic cancer, making this a promising therapeutic approach.^[^ ^7^, ^51^ ^]^ However, the ferroptosis‐mediated suppression of immune cells also facilitate tumor survival, which considerably impedes the utilization of ferroptosis in cancer therapy.^[^ ^9^, ^10^, ^11^, ^12^, ^13^, ^14^ ^]^ Therefore, we aimed to develop a strategy that could effectively induce ferroptosis in tumors and simultaneously stimulate antitumor immune responses.

**Figure 9:**
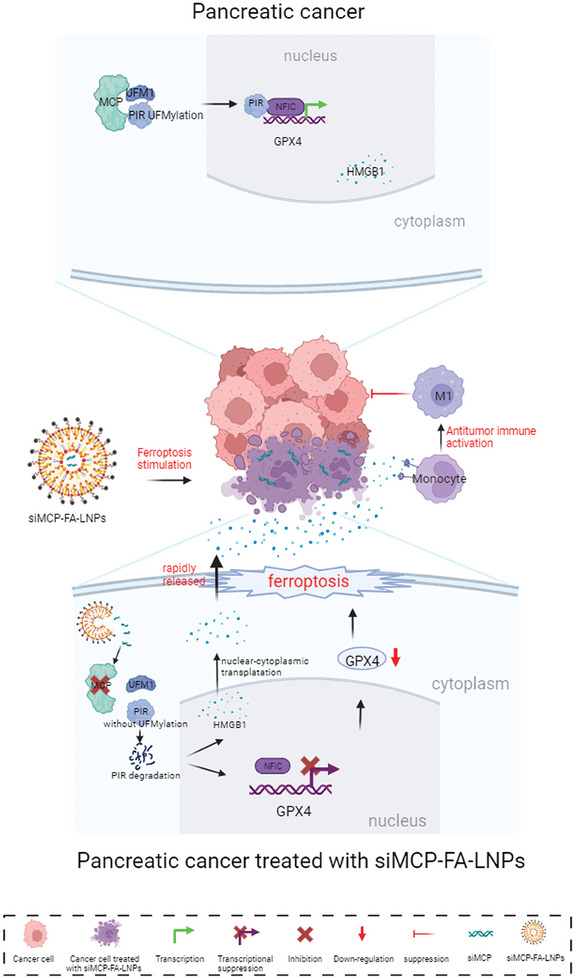
Proposed mechanism of targeting MCP triggers immunogenic ferroptosis in pancreatic cancer. si*MCP*‐FA‐LNPs precisely deliver si‐*MCP* to pancreatic cancer cells, and the resulting knockdown of MCP reduces UFMylation and accelerates the degradation of PIR. This reduces the transcription of GPX4 and promotes the cytoplasmic transportation of HMGB1, ultimately leading to the early and intense release of pre‐accumulated cytoplasmic HMGB1 from the GPX4‐deficient ferroptotic PDAC cells. This altered release pattern of HMGB1 activate the pro‐inflammatory M1‐like macrophages. The dual effects of ferroptosis and tumoricidal pro‐inflammatory M1‐like macrophages effectively suppress pancreatic cancer.

In the present study, we identified a novel target, *MCP*, for the effective activation of ferroptosis and antitumor immune responses. MCP acts as a regulator by enhancing the interaction of UFM1 with PIR, thereby promoting UFMylation of PIR and ultimately stabilizing the protein. By regulating PIR expression, MCP exerts dual biological effects: it induces ferroptosis in pancreatic cancer cells by suppressing *GPX4* and promotes macrophage pro‐inflammatory M1‐like polarization by enhancing cytoplasmic translocation and the early release of HMGB1(**Figure** **9**). Thus, targeting of MCP may result in favorable therapeutic efficacy in PDAC.

We demonstrated that the expression of ferroptosis‐related genes and immune infiltration status were crucial for the prognosis of pancreatic cancer. By stratifying patients based on the expression of ferroptosis‐related genes and their immune infiltration status, we identified that macrophages highly infiltrated the tumors of patients with PDAC and played a significant role in ferroptosis‐related prognosis. In our in vitro, in vivo, and PDO‐based experiments, the tumors tended to stimulate the polarization of macrophages toward the pro‐resolution M2‐like type, as cancer cells can produce numerous products, including extracellular matrix components, interleukin‐10, colony‐stimulating factor‐1, and chemokines such as CCL2, CCL18, CCL17, and CXCL4.^[^ ^52^, ^53^, ^54^, ^55^ ^]^ Stimulation of ferroptosis with RSL3 in pancreatic cancer cells did not alter pro‐resolution M2‐like macrophage polarization. However, activation of ferroptosis by MCP resulted in significant pro‐inflammatory M1‐like polarization both in vivo and in vitro, as well as in PDO models. Results from the orthotopic and subcutaneous transplantation models of NSG and C57BL/6 mice showed that the antitumor efficacy of targeting MCP was superior to that of using RSL3. The poor pharmacokinetics and off‐target effects of RSL3 may weaken its effects as an inducer of ferroptosis in vivo.^[56]^ However, in agreement with the results of other studies,^[^ ^13^, ^57^, ^58^ ^]^ we observed that RSL3 triggered ferroptosis in vivo, which included the suppression of GXP4 and increase in MDA and 4‐HNE levels in IHC. More importantly, by depleting macrophages in C57BL/6 mice and supplementing macrophages in NSG mice, we confirmed that tumoricidal pro‐inflammatory M1‐like macrophages, stimulated by MCP inhibition, and cancer‐promoting pro‐resolution M2‐like macrophages, stimulated by RSL3, were the primary factors contributing to the superior therapeutic efficacy of MCP targeting over that of RSL3. In addition, we generated LNPs for packaging the interfering RNA targeting MCP to ensure the stability of the interfering RNA during transportation in vivo. The FA on the surface of the LNPs ensured precise delivery of the interfering RNA into the tumor, achieving accurate and efficient targeting of MCP in pancreatic cancer cells.

DAMPs, such as ATP, CRT, and HMGB1, promote the recruitment, maturation, and secretion of type I IFN from APCs. Nonetheless, the release of DAMPs by tumor cells undergoing ferroptosis does not guarantee immunogenicity. The specific spatiotemporal pattern of DAMP release is an important factor in the activation of antitumor immune responses.^[59]^ However, the quantity, diversity, and kinetics of DAMP release can vary significantly depending on the activation method used, rendering the achievement of ICD in tumor cells difficult. Consistent with the observations of previous studies,^[60]^ we found that the ferroptotic tumor cells released DAMPs in a specific spatiotemporal pattern, such as the early release of ATP and early exposure of CRT, in cases of both MCP inhibition and RSL3‐induced ferroptosis. However, in contrast to other reports, we found that the timing and amount of HMGB1 released were altered in the MCP‐inhibited cells. HMGB1 is believed to be released at a late stage of ferroptosis, as observed in RSL3‐induced ferroptotic cells. Notably, in the MCP‐inhibited cells, HMGB1 was released in large quantities at the onset of ferroptosis, which was crucial for inducing tumor‐suppressing pro‐inflammatory M1‐like macrophage polarization in our study. We confirmed that the inhibition of MCP‐induced cytoplasmic translocation of HMGB1 was responsible for the altered release pattern, which made MCP a unique inducer capable of triggering immunogenic ferroptosis. This suggests the possibility of activating the immunogenicity of ferroptotic tumor cells by manipulating the pattern of HMGB1 release.

This is the first study to show that MCP can prevent ferroptosis and that the MCP‐PIR axis is a novel pathway that regulates *GPX4* transcription. In particular, we showed that MCP can regulate PIR UFMylation. Recently, UFMylation was found to be closely associated with the occurrence and development of cancer. Here, we demonstrated for the first time the involvement of UFMylation in the regulation of ferroptosis and immunogenicity, indicating the diverse biological functions of UFMylation. UFMylated PIR decreases degradation, thereby increasing protein levels. Thus, highly expressed PIR can act as a transcriptional coactivator that complements the transcription factor, NFIC, to promote GPX4 transcription. Simultaneously, PIR overexpression is also an important regulator of the cytoplasmic translocation of HMGB1.

In the present study, we demonstrated that the changed release pattern of DAMPs in MCP‐deficient pancreatic cancer cells was essential for the polarization of tumoricidal‐polarized pro‐inflammatory macrophages, and that there was upregulation of CD80, CD86, and inducible nitric oxide synthase (iNOS) were up‐regulated in this cohort of macrophages. Considering that iNOS is one of the hallmarks of tumoricidal pro‐inflammatory macrophages, and that the nitric oxide (NO) produced by iNOS exerts anti‐tumor activity,^[61]^ further studies are warranted to investigate the mechanism by which the changed release pattern of DAMPs from the MCP‐deficient tumor cells stimulates the transcription of *iNOS*.

In summary, our findings revealed that suppression of the MCP‐PIR axis resulted in dual antitumor efficacy, triggered ferroptosis in PDAC, and stimulated the polarization of tumoricidal pro‐inflammatory M1‐like macrophages. The nanosystem for targeting MCP developed in this study is an efficient tool for triggering immunogenic ferroptosis and a promising therapeutic for PDAC.

## 4 Experimental Section

### Cell Culture

Human PDAC cell lines and human THP‐1 cell lines were purchased from the American Type Culture Collection (ATCC, VA, USA). BMDMs from C57BL/6 mice were purchased from Procell Life Science & Technology (Procell, Wuhan, China). The Universal Mycoplasma Detection Kit (ATCC) showed these cell lines were not infected with mycoplasma.

### Cell Viability Assay

Cell proliferation was assessed using Cell Counting Kit‐8 (CCK‐8, Dojindo, Kumamoto, Japan), and absorbance was measured spectrophotometrically at 450 nm with a microplate reader (Tecan Trading AG, Switzerland). All absorbance values were normalized to blank wells, and cell viability was normalized to wells treated with DMSO (carrier). The half‐maximal inhibitory concentration (IC_50_) was calculated 48 h after the indicated treatments.

### Cell Death Assay

At 48 h after the indicated treatments, cells were resuspended in PBS and stained using the Cell Cycle and Apoptosis Analysis Kit (PI staining, MCE, NJ, USA) at 37 °C for 30 min. After staining, cells were analyzed using a BD Accuri C6 Plus Flow Cytometer (BD Biosciences, NY, USA). Cell death was normalized to wells treated with DMSO (carrier).

### Organoid Culture

Tumor tissues from patients with PDAC were digested in advanced Dulbecco's modified Eagle's medium (DMEM)/F12 (Gibco, CA, USA) and tissue enzymatic solution I/II (STEMCELL, CA, USA). Advanced DMEM/F12 containing 10% fetal bovine serum (FBS) was used to stop the digestion. The cells were resuspended in Matrigel (Corning, NY, USA). To construct co‐culture models, THP‐1 cells were treated with PMA (80 ng mL^−1^) for 12 h to obtain adherent M0 macrophages, which were then resuspended together with pancreatic cancer cells at a 1:5 ratio in Matrigel. The resuspended cells were plated on 24‐well plates and incubated at 37 °C for 30 min to allow solidification. PancreaCult Organoid growth medium was used for culturing (STEMCELL).

### Differentiation of Macrophages and Co‐Culture Assay

THP‐1 cells (1 × 10^5^ cells mL^−1^) were seeded at the bottom of 6‐well co‐culture plates (Corning 3450) and treated with PMA (80 ng mL^−1^) for 12 h to obtain adherent M0 macrophages. The supernatant and undifferentiated THP‐1 cells were washed with PBS. Tumor cells that had undergone the indicated treatments were seeded in the upper level of the co‐culture plates and co‐cultured with macrophages at a 5:1 ratio for 24 h. The co‐culture of THP‐1 cells and organoids has been described in the previous section. After co‐culturing with tumor cells in vivo, the macrophages were resuspended to obtain single‐cell suspensions. After co‐culturing with tumor cells from the PDO models, macrophages and cancer cells were resuspended and incubated with FITC‐CD68 to isolate macrophages using flow cytometry. The macrophages were incubated with a human FcR‐blocker (BioLegend, CA, USA) for 15 min at 4 °C and then stained with PE‐CD86 (eBioscience, CA, USA) and APC‐CD163 (eBioscience, CA, USA) for 30 min at 4 °C. After staining, the cells were analyzed using a BD Accuri C6 Plus flow cytometer. The FlowJo software was used to analyze the data. The phenotype of macrophages were defined according to the following markers: pro‐inflammatory M1‐like macrophages were CD86^+^/CD163^−^ in flow cytometry and had expression of CD80, CD86, and iNOS measured by RT‐qPCR, pro‐resolution M2‐like macrophages were CD86^−^/CD163^+^ in flow cytometry and had expression of CD163, CD206, and Arg‐1 measured by RT‐qPCR.

### In Vivo Models

All animal experiments were performed in accordance with the guidelines for the Protection of Experimental Animals (http://www.aaalac.org) and was approved by the Animal Protection and Use Committee of Fujian Hospital. C57BL/6 mice were purchased from Slac Laboratory Animal Co., Ltd. (Shanghai, China), and KPC and NSG mice were purchased from Jackson Laboratories (JAX, CA, USA). After tumorigenesis, tumor tissues obtained from the KPC mice were resuspended as single‐cell suspensions. In total, 2 × 10^6^ tumor cells (200 µL) were injected subcutaneously on the right flank of six‐week‐old male NSG or C57BL/6 mice to establish the spontaneous tumor model. In addition, 50 µL tumor cells (1 × 10^8^ cells mL^−1^) were implanted orthotopically in the pancreatic duct of 6‐week‐old male C57BL/6 mice to construct an orthotopic model (stably expressing luciferase). For depleting macrophages, 200 µL clodronate liposomes (Encapsula NanoSciences) were intravenously injected into C57BL/6 mice. To supplement the macrophages, 2 × 10^6^ BMDM cells (100 µL) were co‐injected orthotopically or subcutaneously into NSG mice. For orthotopic models that stably expressed luciferase, tumor growth was visualized using the IVIS system after D‐luciferin injection, and fluorescence intensity was quantified using the total photon flux (photons s^−1^). The tumor volume (mm^3^) was calculated as (L × W^2^)/2, where L represents the longest axis and W the perpendicular axis.

### Lipid Peroxidation Assay

After staining with 10 µm BODIPY‐581/591 C11 (Thermo Fisher Scientific, MA, USA) for 30 min, the cells were analyzed using flow cytometry and fluorescence microscopy. Fluorescence intensity was obtained as follows: (area of the region emitting green fluorescence)/(area of DAPI) ×100. The fluorescent region was identified using the ImageJ software. The fluorescence intensity was normalized to the mean value of three replicates of the control groups.

### Detection of Ferroptosis Associated Indicators

After collecting cell lysates, the MDA level was evaluated by the MDA Assay Kit (Abcam, MA, USA). The concentration of 4‐HNE was analyzed using ELISA kits (MyBioSource, CA, USA), and the GSH/GSSG ratio was determined using the GSH/GSSG Kit (Beyotime, Shanghai, China).

### RNA Isolation and RT‐qPCR Analysis

Total RNAs from PDAC tissues or cell lines were extracted using the TRIzol Reagent (Invitrogen, CA, USA). Reverse transcription was conducted with the PrimeScript RT Reagent Kit (Takara, Dalian, China). The StepOnePlus Real‐Time PCR System (Termo Fisher Scientifc, MA, USA) was used to perform the real‐time PCR reactions. The relative mRNA level was normalized to *GAPDH* using the 2^−△△CT^ method.

### Western Blotting

Proteins from PDAC tissues or cell lines were extracted using a RIPA buffer (Solarbio, Beijing, China). Proteins from the nucleus or cytoplasm were isolated using the Nuclear and Cytoplasmic Extraction Kit (Thermo Fisher Scientific, MA, USA). The concentration of proteins was measured using the BCA method (Beyotime, Beijing, China). Proteins were separated on SDS‐polyacrylamide gels, and then transferred to polyvinylidene fluoride membranes. The membranes were blocked in 5% skim powdered milk for 1 h, and then incubated with primary antibodies. After washing off the unbound primary antibodies, the membranes were incubated with secondary antibodies. The targeted proteins were detected using Pierce ECL Reagent (Thermo Fisher Scientific, MA, US).

### Detection of UFMylation

Cells were lysed after boiling in a strong denaturant containing 150 mM Tris‐HCl, 30% glycerol, and 5% SDS to remove noncovalently bound of UFM1. The lysates were then diluted by the dilution buffer (50 mm Tris‐HCl, 150 mm NaCl, 10 mm imidazole, 1% Triton X‐100 and 2 mm NEM). Immunoprecipitation was performed using the supernatants.

### Measurement of DAMPs

After collecting supernatants, the levels of HMGB1 were evaluated using an ELISA kit (IBL‐International, Hamburg, Germany). ATP was measured using the Luminescent Cell Viability Assay kit (Promega, WI, USA) and TNFα and IFNγ were measured using ELISA kits (Abcam, MA, USA). For the analysis of calreticulin exposure, cells were collected and incubated with anti‐calreticulin specific antibody (Abcam) for 30 min. Then the cells were washed three times with FACS buffer and stained with secondary FITC antibody (Thermo Fisher Scientific) for 30 min. The cells were analyzed using a BD Accuri C6 Plus flow cytometer. The FlowJo software was used to analyze the data.

### Immunofluorescence

After fixation in 4% paraformaldehyde at room temperature for 15 min, cells were stained with a rabbit anti‐HMGB1 primary antibody (Cat. No. ET1601‐2, HUABIO) overnight at 4 °C. After washing with PBS three times, the cells were incubated with CY3 goat anti‐rabbit secondary antibody (Cat. No. GB21303, Servicebio, Wuhan, China). The nucleus was stained by DAPI (ab104139, Abcam). Cells were observed using a laser scanning confocal microscope (Nikon Instruments Inc., Japan).

### Immunohistochemistry and H&E Staining

IHC staining of tissue sections were independently evaluated by two experienced pathologists. The staining intensity score was divided into 0 (negative), 1 (weak), 2 (moderate), or 3 (strong). The score for number of positive tumor cells was classified as 1 (0–25%), 2 (26–50%), 3 (51–75%), or 4 (76–100%). The total IHC score was calculated by multiplying the scores for staining intensity and positivity. The positive percentage was calculated using the ‘Trainable Weka Segmentation’ in Image J software, a plugin that can recognize the positively or negatively stained cells, according to the previous study.^[62]^ The sections of hearts, livers, spleens, lungs, and kidneys were stained with hematoxylin‐eosin (H&E).

### Preparation of LNPs

The lipid mixture consisted of DSPE‐PEG2000, DSPC, cholesterol, and SM‐102 (in a ratio of 28.5:10:1.5:50) was combined with siRNA (in a ratio of 1:2). The lipid nanoparticles were concentrated using a 100 kDa molecular weight cutoff (MWCO) membrane. Dynamic light scattering (DLS) and the polydispersity index (PDI) of LNPs were analyzed by Zeta Sizer (NanoZS, Malvern, U.K.). The encapsulation efficiency of siRNA in LNPs was measured from the level of fluorescence with the RiboGreen kit (Invitrogen, CA, USA) before ultra‐filtration. The siRNA encapsulation efficiency was calculated as: (F1− F2)/F1 × 100%, where F1 and F2 are fluorescence levels with and without Triton X‐100, respectively. To determine the delivery efficiency of LNPs, cells were incubated with free‐Cy5‐si*MCP*, RNAiMAX‐Cy5‐siMCP, or Cy5‐ siMCP‐LNPs (concentration of Cy5‐si*MCP* was 50 nm in all groups) for 48 h. The cells were then lysed and the fluorescence of Cy5 was measured by SpectraMax iD5 microplate reader (Molecular Devices, CA, USA).

### Biodistribution of LNPs

C57BL/6 mice with subcutaneous tumors were injected with saline, free Cy5‐siRNA, or Cy5‐siRNA‐FA‐LNPs (2.5 mg siRNA kg^−1^) via the tail vein. The tumors and major organs were harvested 48 h after the injection. Fluorescence images were acquired using the IVIS Spectrum imaging system (PerkinElmer, MA, USA) and fluorescence intensity was quantified using total radiance (p per s per cm^2^ per sr).

### Agarose Gel Electrophoresis

Nucleic acid samples were loaded onto 2% (w/v) agarose gel, followed by separation utilizing electrophoresis in a Tris‐acetate EDTA running buffer at a voltage of 120 V for 30 min. Gel images were subsequently visualized utilizing the ChemiDoc MP Imaging System (Bio‐Rad, CA, USA).

### scRNA‐Seq

Three weeks after injection, tumors from the C57BL/6 orthotopic model were harvested and digested using collagenase II and IV (0.25%), and DNase I (0.05%) in DMEM/F12 at 37 °C for 30 min. Cancer cells (1.5 × 104 in each tumor) were loaded onto the 10X Genomics Chromium platform. Samples were processed using Single Cell 3′ v2 reagent and then sequenced using an Illumina NextSeq sequencer. Cellranger 3.1.0 was used to map raw data to the mouse genome, mm10‐3.0.0. The Seurat R package was used for unsupervised clustering. Genes expressed in fewer than two cells were excluded. Cells with more than 200 genes, less than 10% of which were mitochondrial, were processed further. Subsequently, the variation coefficient of the genes was determined using the Seurat R package, and dimensionality reduction was performed using principal component analysis based on the top 2,000 most variable genes. The Euclidean distances of the top ten significant principal components in space were used to construct the k‐nearest neighbor graph. Cells in the graph were clustered using the Louvain Modularity optimization algorithm and then visualized using the t‐distributed Stochastic Neighbor Embedding (tSNE) project. The annotation of these clusters was based on typical markers of previously identified cell types in the CellMaker‐2.0 database.^[63]^

### Generation of Ferroptosis‐Related Risk Score and Immune‐Related Risk Score

All the genes in the FerrDb were collected, including those defined as promoters or suppressers of ferroptosis, or those possibly related to ferroptosis. Based on the expression of ferroptosis‐related genes from FerrDb (http://www.zhounan.org/ferrdb), consensus clustering was performed using the ConsensusClusterPlus package in R. Ferroptosis‐related genes were first analyzed using univariate Cox regression. Then, LASSO regression analysis of ferroptosis‐related genes and determination of prognostic values was used to obtain coefficients for all genes. The risk score for each sample was equal to the sum of gene expression multiplied by the corresponding coefficient. Based on the median risk score, the TCGA‐PADC samples were divided into high‐risk and low‐risk groups. Analysis of overall survival (OS) was performed to identify the prognostic value of the signature. The estimate package in R was used to determine to the immune score of each patient.

### Establishment of the Combined‐Risk Score Model

Patients with a high ferroptosis risk score and a high immune risk score were classified into the combined‐risk score High group (CRS‐H), patients with a low ferroptosis risk score and a low immune risk score were classified into the combined‐risk score Low group (CRS‐L). The limma package in R was used to identify differentially expressed genes (DEGs) by measurement of fold‐change (FC) in these two subtypes, based on specific criteria (threshold: |log_2_(FC)| > 1, adjusted P < 0.05). LASSO regression was performed to obtain model genes and develop a nomogram for clinical validation. The precision of the 5‐year survival rates predicted by the ferroptosis risk score model, the immune risk score model, and the CRS model were evaluated with time‐dependent receiver‐operating characteristic (ROC) curve analysis using the timeROC package in R.

### Functional Analysis and Assessment of Immune Cell Infiltration

The clusterProfiler package in R was used for Gene Ontology (GO) and Kyoto Encyclopedia of Genes and Genomes (KEGG) analyses, based on specific criteria (DEG: (|log_2_(FC)| > 1, FDR <0.05) that were obtained by comparison of the different groups. The Goplot package in *R* was used to construct these plots. Specific criteria (|log_2_(FC)| > 2, adjusted *P* < 0.05) were used to identify significant differences. The adjusted P‐value was calculated using the Benjamini‐Hochberg multiple test correction. Differences in expression were determined using the limma package in R.

### Assessment of the Immune Microenvironment Landscape

The CIBERSORT package in R was used to evaluate the proportions of 22 different immune cells and interpret the immune environment of the ferroptosis‐immune related group. The simulation was performed with 1000 replicates to determine the composition of these 22 immune cells in each sample, and the limma and pheatmap packages in *R* were used to analyze differences in immune cell infiltration between the high‐risk and low‐risk groups. A P value below 0.05 was considered significant. The markers from 22 immune cells, including innate immune cells, adaptive immune cells, endothelial cells, and fibroblasts, were identified in a previous publication.^[64]^ Based on these markers, a single‐sample gene set enrichment analysis (ssGSEA) algorithm was then used to evaluate the extent of infiltration of immune cells in the tumor microenvironment. The differential expression of immune‐related genes in the high‐risk and low‐risk groups was also analyzed using the limma and pheatmap packages in R. A P value below 0.05 was considered significant.

### Construction of the Coexpression Network

Weighted Gene Co‐Expression Network Analysis (WGCNA) was conducted to identify gene sets (modules) that were associated with the 22 kinds of immune cells using the WGNCA package in R.^[65]^ The optimal soft threshold was determined by scale independence and mean connectivity analysis on modules with different power values. The soft threshold was 9 when the signed *R* ^2^ value was 0.9. Next, correlations among gene expression modules and different kinds of immune cells were estimated by calculation of Pearson's correlation coefficients for the module eigengene (ME) of each module and each kind of immune cell. This analysis led to identification of modules that were significantly related to a trait (*P* < 0.05).

### Statistical Analysis

GraphPad Prism 9.0 and SPSS 23.0 were used for statistical analyses. For relative values, the data of each group were normalized to the mean value of the three replicates of the control group. Data are presented as mean ± standard error of mean (SEM) unless otherwise indicated. Sample size (*n*) for each statistical analysis is mentioned in the legends of corresponding graphs. Statistical comparisons of continuous variables between two groups were performed using Student's *t*‐test, and the Mann‐Whitney U test was used when the data had non‐normal distribution. Comparisons of multiple groups were performed using analysis of variance (ANOVA). Overall survival and progression‐free survival were assessed using the Kaplan−Meier method, and survival curves were compared using the log‐rank test. Pearson's χ2 test or Fisher's exact test was used to analyze the categorical variables. P‐values less than 0.05 were considered statistically significant.

### Ethical Approval

The ethical approval of human research and animal experiments in our study was obtained from the Animal Care and Use Committee of Fujian Provincial Hospital Institutional, the assigned approva number is K2021‐03‐046 for human research, and IACUC FJABR 2022060101 for animal experiments.

## Conflict of Interest

The authors declare no conflict of interest.

## Author Contributions

G.L., C.L., and J.C. contributed equally to this work. S.C. and Y.C. designed the research and directed the work. G.L. and C.L. performed the experiments and wrote the paper. J.C., S.Z., and Z.W. participated in data analysis and collected the tissues. Y.C., D.W., J.L., and Y.T. provided technical help. All authors read and approved the final manuscript.

## Supporting information

Supporting Information

## Data Availability

The data that support the findings of this study are available from the corresponding author upon reasonable request.
