# Supplementary material for: Targeting the MCP‐GPX4/HMGB1 Axis for Effectively Triggering Immunogenic Ferroptosis in Pancreatic Ductal Adenocarcinoma
Source: Adv Sci (Weinh). 2024 Apr 9;11(21):2308208. doi: 10.1002/advs.202308208 (PMC11151063; doi:10.1002/advs.202308208)
Supplement: Supplementary file 1 — Supporting Information [file ADVS-11-2308208-s001.pdf]

ADVANCED SCIENCE
Open Access

Supporting Information

for *Adv. Sci.*, DOI 10.1002/advs.202308208

Targeting the MCP-GPX4/HMGB1 Axis for Effectively Triggering Immunogenic Ferroptosis in Pancreatic Ductal Adenocarcinoma

Ge Li, Chengyu Liao, Jiangzhi Chen, Zuwei Wang, Shuncang Zhu, Jianlin Lai, Qiaowei Li, Yinhao Chen, Dihan Wu, Jianbo Li, Yi Huang, Yifeng Tian, Yanling Chen and Shi Chen**

Supplementary Figure Legends

Supplementary FIG. 1.

A, Unsupervised clustering analysis (k = 3) of patients with pancreatic cancer (TCGA) based on the expression of ferroptosis-related genes. **B**, Kaplan–Meier analysis of overall survival based on the immune risk subtype and ferroptosis risk subtype. **C**, Kaplan–Meier analysis of overall survival based on a combination of immune and ferroptosis risk subtypes. **D**, DEGs between the two CRS groups. **E,** Expression of MHC classes, costimulators, and coinhibitors in the CRS groups. **F,** Immune cell signature scores in the CRS groups. **G**, Abundance of immune cells in the tumor microenvironment of CRS-H and CRS-L groups. **H**, Weighted gene co-expression network analysis (WGCNA) of CRS-DEGs and immune cells. The module eigengene (ME) values were correlated with binary variables (Spearman's correlation) that represent different types of immune cells. Within each table cell, the upper values represent correlation coefficients between ME and the variable; the P value of the corresponding module trait is shown in the brackets below. **I**, LASSO analysis of DEGs from the CRS groups to screen hub genes. **J**, Multivariate Cox regression analysis of the hub genes and clinical factors. **K**, Nomogram constructed based on independent factors from the multivariate Cox regression analysis. **L,** *MCP* mRNA levels in PDAC and normal pancreatic tissues (TCGA & GTEx). **M**, Overall survival and disease-free survival of patients with PDAC based on *MCP* expression (TCGA). Data are represented as the mean ± SEM. P values are presented as ns P > 0.05, ** P < 0.01, and ***P < 0.001. One-way ANOVA (G, L), and log-rank tests (B, C, and M) were used.

Supplementary FIG. 2.

A, *MCP* mRNA level in PDAC cell lines (n = 3). **B**, *MCP* mRNA level in PANC-1 cells transfected with si-*NC* or si-*MCP,* and in PaTu8988t cells after transfection with OE-*CTRL* or OE-*MCP* (n = 3). **C**, Proliferative activity of si-*NC*/si-*MCP*-PANC-1 cells and OE-*CTRL*/OE-*MCP*-PaTu8988t cells grown for 5 days as assessed by CCK8 (n = 3). **D**, *MCP* mRNA level in MIA PaCa-2 cells and BxPC-3 cells transfected with OE-*CTRL* or OE-*MCP*, and in Capan-2 cells transfected with si-*NC* or si-*MCP* (n = 3). **E**, Proliferative activity of OE-*CTRL*/OE-*MCP*-MIA PaCa-2 cells, and OE-*CTRL*/OE-*MCP*-BxPC-3 cells, and si-*NC*/si-*MCP* Capan-2 cells grown for 5 days as assessed by CCK8 (n = 3). **F-K**, Levels of lipid peroxidation measured by fluorescence microscopy (F-H, scale bar: 30 μm) and flow cytometry (I-K); fluorescence intensity was normalized to the mean value of three replicates of the OE-*CTRL* (F-G, I-J) and si-*NC* (H, K) groups (n = 3). **L-Q**, Levels of GSH/GSSG ratio (L-N) and MDA (O-Q) (n = 3). **R-U**, Expression of ferroptosis-related genes in the indicated cell lines at the mRNA (R-T) and protein (U) levels (n = 3). **V-X**, Expression of MCP and GPX4 proteins (V) and mRNAs (W-X) in OE-*MCP*-PaTu8988t cells transfected with si-*GPX4* (n = 3). n indicates the number of biological replicates. Data are represented as the mean ± SEM. P values are presented as ns P > 0.05, ** P < 0.01, and ***P < 0.001. Unpaired two-tailed Student's t-test (F-T), one-way (A-B, D, W-X) and two-way ANOVA (C, E) were used.

Supplementary FIG. 3.

A, Spearman's correlation analysis between MCP and the infiltration of pro-inflammatory M1-like and pro-resolution M2-like macrophages (TIMER2, http://timer.comp-genomics.org/). **B**, ssGSEA analysis of the correlation between MCP and immune cells in the tumor microenvironment. **C**, Correlation between MCP and the abundance of immune cells in the tumor microenvironment (http://tisch.comp-genomics.org/). **D**, MCP protein level in tumors treated as described in Fig. 3B. **E-F**, Levels of lipid peroxidation measured by fluorescence microscopy (E, scale bar: 30 μm) and flow cytometry (F); fluorescence intensity was normalized to the mean value of three replicates of the OE-*CTRL* groups (n = 3). **G-H**, Levels of GSH/GSSG ratio (G) and MDA (H) in KPC cells (n = 3). **I**, Tumor growth in subcutaneous transplantation models of NSG (n = 5) and C57BL/6 mice (n = 5). **J**, Immunohistochemistry analysis and quantification of the indicated markers in subcutaneous transplantation models from C57BL/6 mice (n = 5); scale bar: 100 μm. **K**, Tumor growth in NSG mice co-inoculated with BMDM and tumor cells (n = 5) and in clodronate liposome-treated C57BL/6 mice (n = 5). **L**, Macrophage depletion by clodronate liposomes was confirmed using flow cytometry (n = 3). **M-N**, mRNA levels of pro-inflammatory M1-like and pro-resolution M2-like markers were analyzed in macrophages co-cultured with the indicated PDO models (M) and PDAC cell lines (N). n indicates the number of biological replicates (E-H, M-N) or individual mice (I-L). Data are represented as the mean ± SEM. P values are presented as ns P > 0.05, ** P < 0.01, and ***P < 0.001. Unpaired two-tailed Student's t-test (K) and one-way (E-J, L-N) ANOVA were used.

Supplementary FIG. 4.

A, *FOLR1* mRNA levels in PDAC and normal pancreatic tissues (TCGA & GTEx). **B**, Representative ex vivo fluorescence images and statistical analyses of tumors and major organs from tumor-bearing C57BL/6 mice (n = 3). **C-D**, Safety of LNPs was confirmed by hematoxylin-eosin staining (C, scale bar: 100 μm) and by measuring body weight (D) (n = 3). **E**, Procedure for the transfer of tumor cells from KPC mice into NSG and C57BL/6 mice; RSL3 (40 mg/kg) or si*MCP*-FA-LNPs (siMCP 2.5 mg/kg) was intravenously injected qod. **F**, MCP protein levels in tumors that received treatment, as described in Fig. S4E. **G**, Tumors were harvested from the mice 16 days after the first treatment, tumor volume were measured (n = 5). **H**, Overall survival time of C57BL/6 mice with the indicated treatments (n = 5). **I**, Representative images and statistical analysis of immunohistochemistry in C57BL/6 mice. Scale bar: 100 μm (n = 5). n indicates individual mice (B-D, G-I). Data are represented as the mean ± SEM. P values are presented as ns P > 0.05, ** P < 0.01, and ***P < 0.001. One-way (A, G and I) and two-way (B, D) ANOVA and Log-rank test (H) were used.

Supplementary FIG. 5.

A-B, IFN-γ and TNF-α levels in the supernatants of control, ferroptosis-resistant, and ferroptotic groups in PANC-1 cells (n = 3). **C-D**, Time-dependent changes in IFN-γ and TNF-α levels in the supernatant of PANC-1 cells following the indicated treatments (n = 3). **E**, mRNA levels of

HMGB1 in PANC-1 cells following the indicated treatments (n = 3). **F**, Protein levels of MCP, HMGB1, and ferroptosis-related genes in PANC-1 cells after treatment with leptomycin B (50 ng/mL for 12 h). **G**, RT-qPCR analysis of the markers of pro-inflammatory M1-like and pro-resolution M2-like phenotypes in macrophages that were co-cultured with leptomycin B-treated si-*MCP* PANC-1 cells (n = 3). **H-I**, Levels of lipid peroxidation measured by flow cytometry (H) and fluorescence microscopy (I, scale bar: 30 μm) ; fluorescence intensity was normalized to the mean value of three replicates of the si-*NC* groups (n = 3). **J-K**, Levels of GSH/GSSG ratio (J) and MDA (K) in si-*MCP* PANC-1 cells treated with or wihout leptomycin B (n = 3). n indicates the number of biological replicates. Data are represented as the mean ± SEM. P values are presented as ns P > 0.05, ** P < 0.01, and ***P < 0.001. One-way (A-B, E, G-K) and two-way (C-D) ANOVA were used.

Supplementary FIG. 6.

A, Relative mRNA levels of *PIR*, *PKC*, *CaMK4*, *STAT1*, and *HSP72* in si-*MCP*-PANC-1 cells and OE-*MCP*-PaTu8988t cells (n = 3). **B**, Protein-protein interactions among PKC, CaMK4, STAT1, HSP72, PIR, and MCP (STRING, www.string-db.org/). **C**, Relative mRNA levels of *HMGB1* in the si-*PIR*-PaTu8988t and OE-*PIR*-PANC-1 cells (n = 3). **D-E**, Changes in protein levels measured in Fig. 6C and D, were quantified using line charts (n = 3). **F**, Protein-protein docking of UFM1 and PIR (BioLuminate simulation). **G**, Changes in protein levels measured in Fig. 6L, were quantified using line charts (n = 3). **H**, mRNA levels of *HMGB1* in OE-*MCP*-PaTu8988t cells transfected with si-*UFM1* (n = 3). **I**, A consistent UFMylation site in PIR. **J**, Changes in protein levels measured in Fig. 6O, were quantified using line charts (n = 3). n indicates the number of biological replicates. Data are represented as the mean ± SEM. P values are presented as ns P > 0.05, ** P < 0.01, and ***P < 0.001. Unpaired two-tailed Student's t-test (C), one-way (A, H) and two-way ANOVA (D-E, G, and J) were used.

Supplementary FIG. 7.

A-B, Levels of lipid peroxidation in OE-*MCP*-PaTu8988t cells transfected with si-*PIR* (A), and in si-*MCP*-PANC-1 cells transfected with *PIR* (B) (n = 3); scale bar: 30 μm. Fluorescence intensity was normalized to the mean value of three replicates of the OE-*CTRL* groups (A) or si-*NC* groups (B). **C**, Possible NFIC-binding sites in the *GPX4* promoter according to JASPAR. **D**, Primers for the predicted binding region in the *GPX4* promoter used in the ChIP assay. **E-F**, Chromatin immunoprecipitation showing the binding of NFIC and PIR to the promoter of *GPX4* in OE-*PIR* and OE-*NFIC*-PANC-1 cells, respectively (n = 3). n indicates the number of biological replicates. Data are represented as the mean ± SEM. P values are presented as ns P > 0.05, ** P < 0.01, and ***P < 0.001. One-way ANOVA (A-B, and E-F) were used.

Supplementary Figures

Supplementary Figure 1

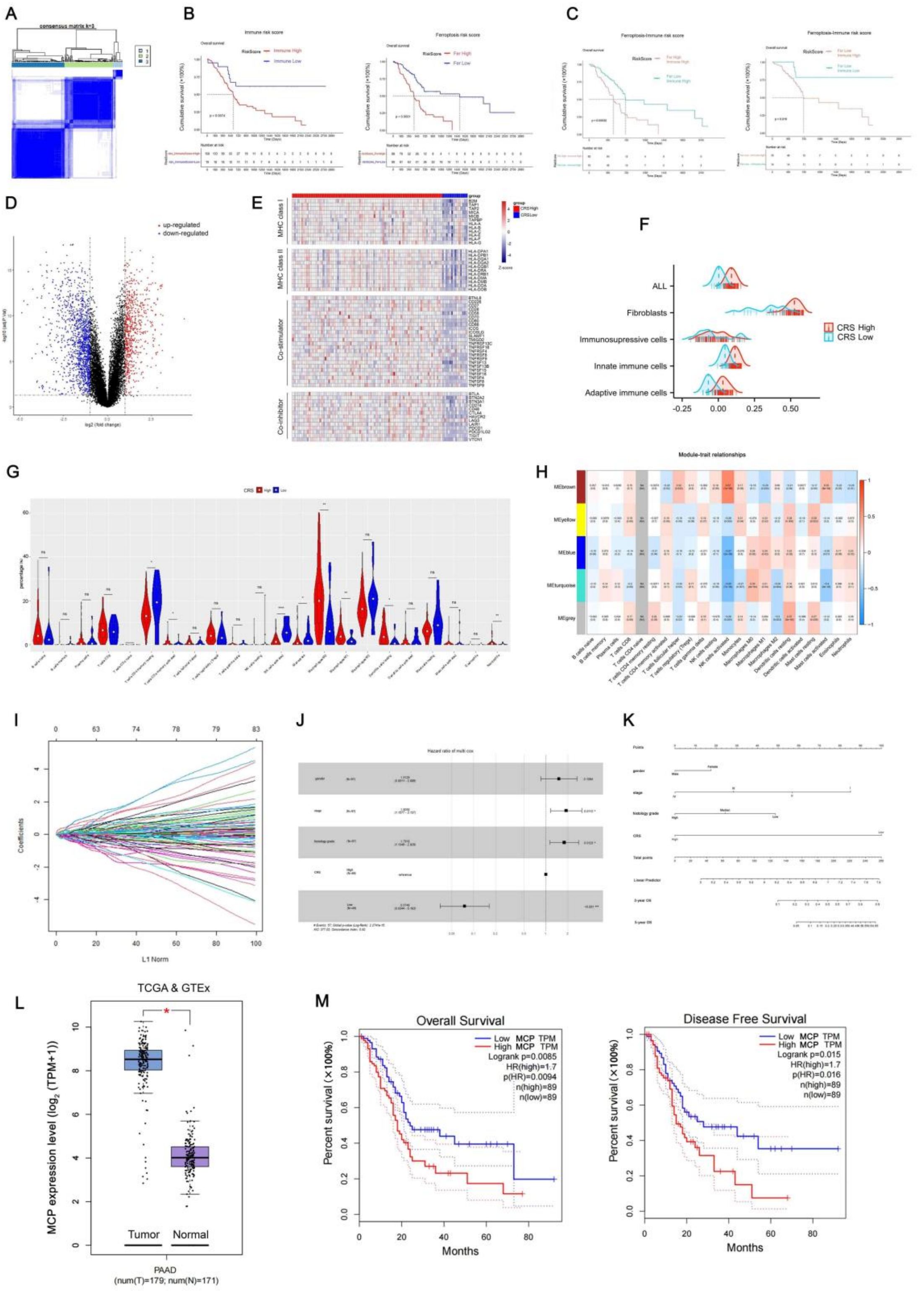

Supplementary Figure 2

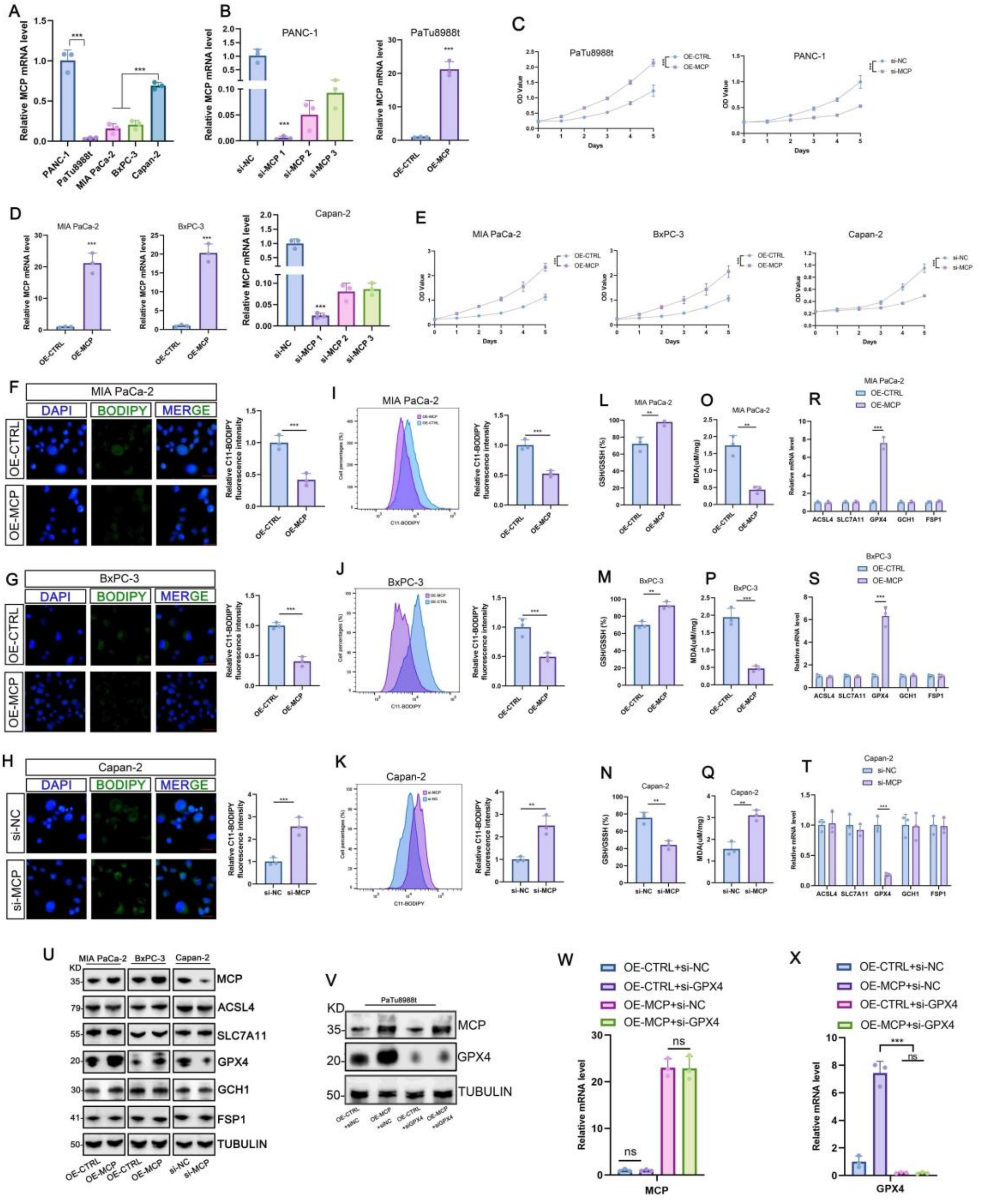

Supplementary Figure 3

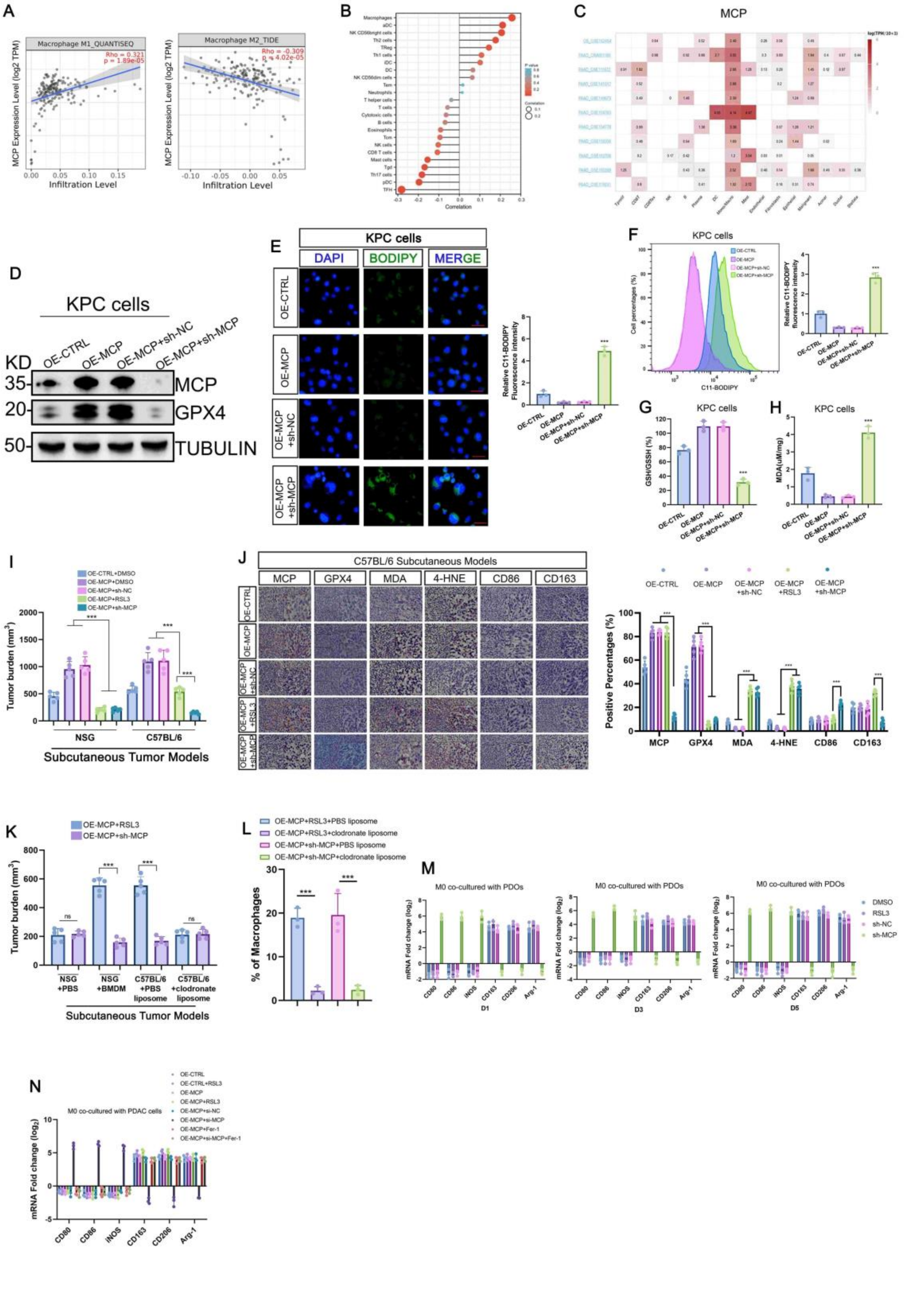

Supplementary Figure 4

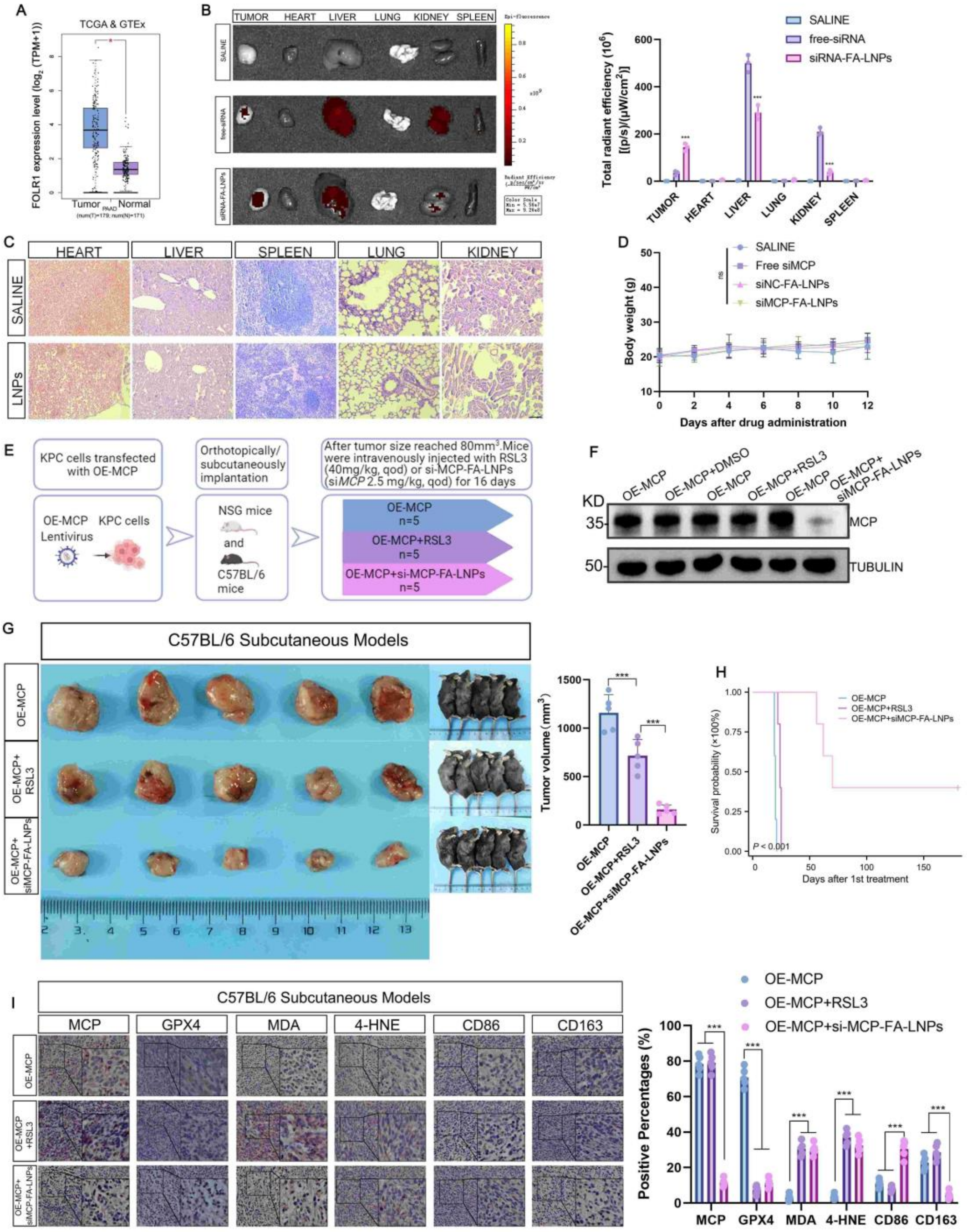

Supplementary Figure 5

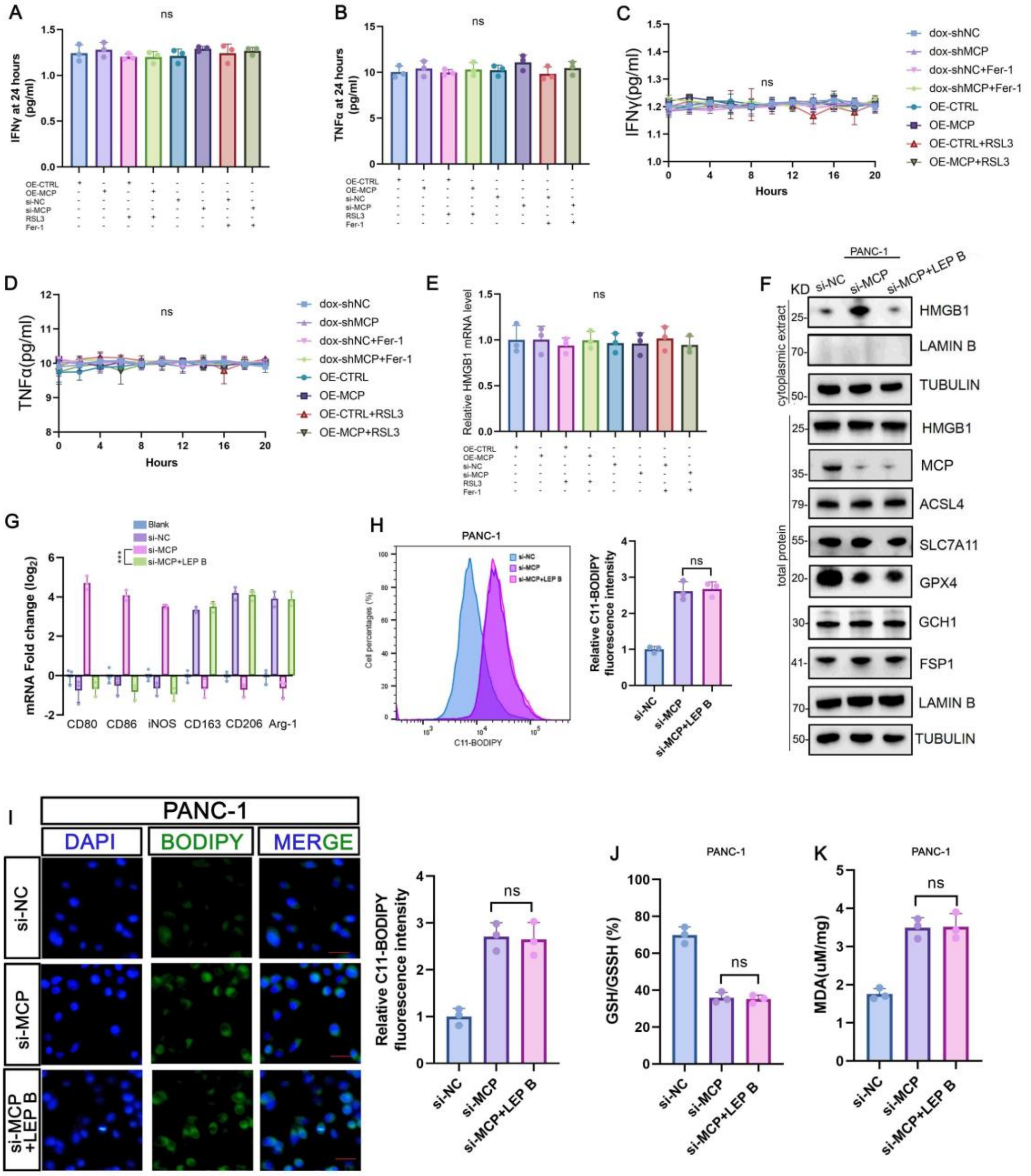

Supplementary Figure 6

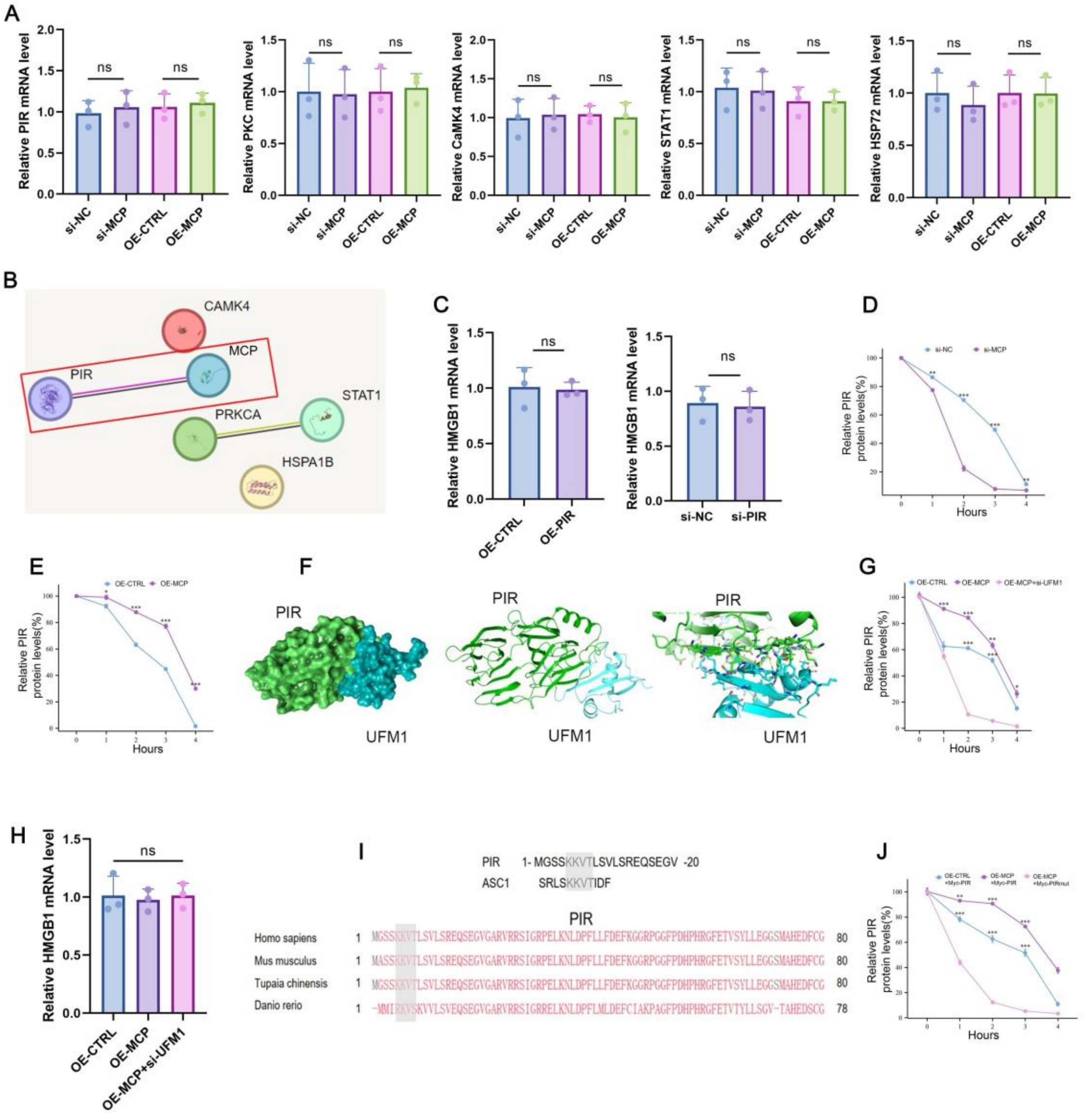

Supplementary Figure 7

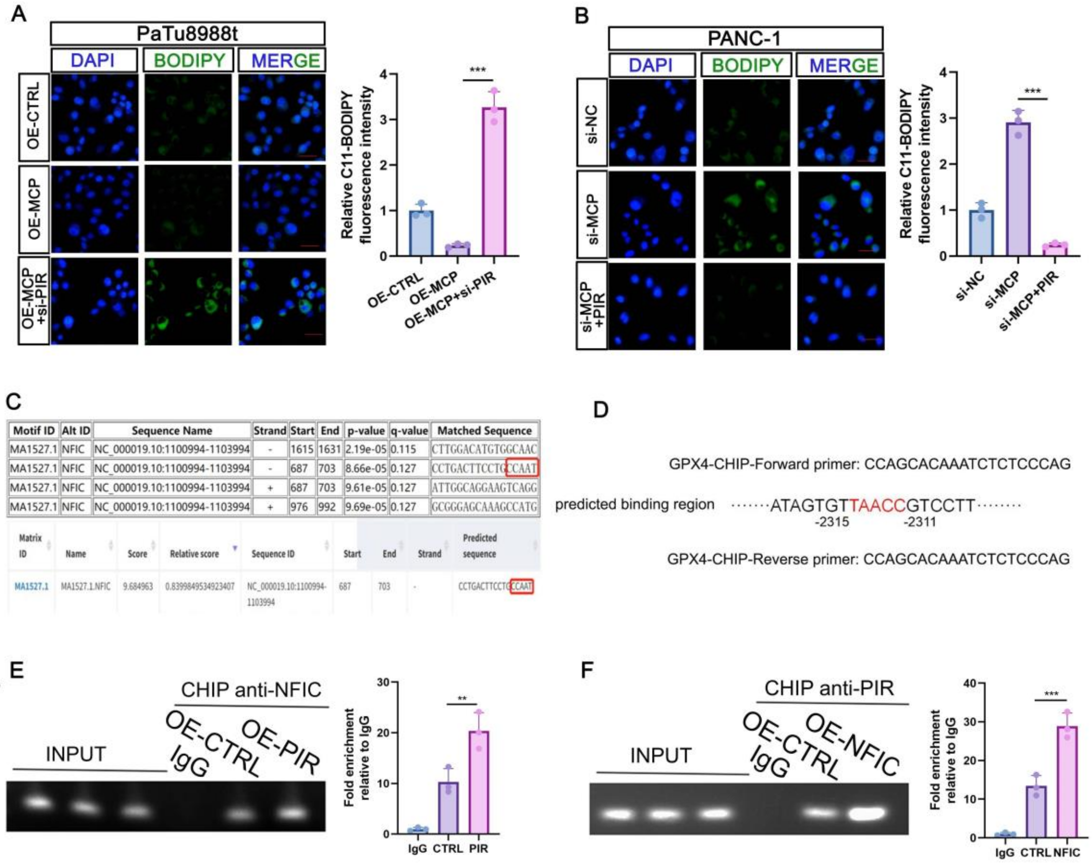

Motif ID	Alt ID	Sequence Name	Strand	Start	End	p-value	q-value	Matched Sequence
MA1527.1	NFIC	NC_000019.10:1100994-1103994	-	1615	1631	2.19e-05	0.115	CTTGGACATGTGGCAAC
MA1527.1	NFIC	NC_000019.10:1100994-1103994	-	687	703	8.66e-05	0.127	CCTGACTTCCTGCCAAT
MA1527.1	NFIC	NC_000019.10:1100994-1103994	+	687	703	9.61e-05	0.127	ATTGGCAGGAAGTCAGG
MA1527.1	NFIC	NC_000019.10:1100994-1103994	+	976	992	9.69e-05	0.127	GCGGGAGCAAAGCCATG

Matrix ID	Name	Score	Relative score	Sequence ID	Start	End	Strand	Predicted sequence
MA1527.1	MA1527.1.NFIC	9.684963	0.8399849534923407	NC_000019.10:1100994-1103994	687	703	-	CCTGACTTCCTGCCAAT
